# Physicochemical characteristics and toxicity of surface-modified zinc oxide nanoparticles to freshwater and marine microalgae

**DOI:** 10.1038/s41598-017-15988-0

**Published:** 2017-11-21

**Authors:** Mana M. N. Yung, Paul-Antoine Fougères, Yu Hang Leung, Fangzhou Liu, Aleksandra B. Djurišić, John P. Giesy, Kenneth M. Y. Leung

**Affiliations:** 1 The Swire Institute of Marine Science and School of Biological Sciences, the University of Hong Kong, Pokfulam, Hong Kong, China; 2 Université de Bordeaux, Bordeaux, France; 3 Department of Physics, the University of Hong Kong, Pokfulam, Hong Kong, China; 4 Department of Veterinary Biomedical Sciences and Toxicology Centre, University of Saskatchewan, Saskatoon, SK Canada; 5 State Key Laboratory of Pollution Control and Resource Reuse, School of the Environment, Nanjing University, Nanjing, China; 6 State Key Laboratory in Marine Pollution, City University of Hong Kong, Tat Chee Avenue, Kowloon, Hong Kong, China

## Abstract

Because of wide applications of surface-modified zinc oxide nanoparticles (ZnO-NPs) in commercial sunscreens and their easiness of being released into water, concerns have been raised over their potential effects on aquatic organisms. This study compared physicochemical properties of silane-coated and uncoated ZnO-NPs to elucidate their toxic potencies toward three freshwater and three marine microalgae. Surfaces of ZnO-NPs (20 nm) were modified by coating with 3-aminopropyltrimethoxysilane (A-ZnO-NPs) that provides the particles with a more hydrophilic surface, or dodecyltrichlorosilane (D-ZnO-NPs) that turns the particles to hydrophobic. Uncoated ZnO-NPs formed larger aggregates and released more Zn^2+^ than did either of the two coated ZnO-NPs. The three nanoparticles formed larger aggregates but released less Zn^2+^ at pH 8 than at pH 7. Although sensitivities varied among algal species, A-ZnO-NPs and uncoated ZnO-NPs were more potent at inhibiting growth of algal cells than were D-ZnO-NPs after 96-h exposure to ZnO, uncoated ZnO-NPs, each of the coated ZnO-NPs or ZnSO_4_ at 10 concentrations ranging from 0.1 to 100 mg/L. The marine diatom *Thalassiosira pseudonana* exposed to ZnO-NPs, A-ZnO-NPs or D-ZnO-NPs resulted in differential expressions of genes, suggesting that each of the coatings resulted in ZnO-NPs acting through different mechanisms of toxic action.

## Introduction

Due to their wide band gap of 3.37 eV at room temperature, zinc oxide nanoparticles (ZnO-NPs), with at least one dimension between 1 and 100 nm, are excellent absorbers of ultraviolet radiation of solar light^1^ and hence are widely used in sunscreens^2^ and photocatalysts^3–5^. Moreover, because ZnO-NPs can induce oxidative stress in cells, they can be applied as antibacterial agent and anti-cancer drug^6–9^. For example, various structures of ZnO-NPs such as nano-rods, nano-sheets, nano-flowers can inhibit growth of bacteria *Escherichia coli*, *Staphylococcus aureus and Klebsiella pneumoniae* ^8^. ZnO-NPs at 100 mg/L can inhibit formation of biofilm by generating reactive oxygen species (ROS), and such an antibacterial activity was optimum at pH 7^6^. ZnO tetrapods can act as antifouling agent to prevent fouling organisms from growing on substrate^7^. As anti-cancer agent, ZnO spherical nanoparticles can induce oxidative stress and apoptosis in liver cancer cells HepG2 and breast cancer cells MCF-7 after 24 h^10^. ZnO quantum dots at 10 µg/mL can control the growth of liver cancer cells HepG2 via ROS production^11^, and ZnO nanorods can induce cell death of C2C12 cancer cells via caspase-dependent pathways^9^. ZnO tetrapods can neutralize HSV-2 virions and serve as a microbiocide to prevent HSV-2 infection, while such antiviral effects can be enhanced under UV light^12,13^.

However, nanoparticles tend to aggregate because of their high surface-to-volume ratio and high surface energy, which hinders their efficacy^14^. One approach to enhance dispersion of nanoparticles is to modify surfaces of particles by coating them with an agent to increase steric or electrostatic repulsion between nanoparticles^15^. Modifications of surfaces can affect physicochemical properties and hence alter toxic potency of ZnO-NPs^16^, gold nanoparticles^17^ or silver nanoparticles^18^ to different extents. Organosilanes, a common coating agent for ZnO-NPs used in sunscreens, can form strong and stable covalent bonds with surfaces of ZnO-NPs and create a shielding barrier of cross-linked polysiloxanes that prevent the core ZnO-NPs from decomposition, aggregation and agglomeration^19,20^. Coating with organosilanes can make nanoparticles more stable by reducing their photocatalytic activities of ZnO-NPs^21^.

Because the coating serves as a barrier between nanoparticles and the environment, and hinders generation of ROS, ZnO-NPs coated with HP1 (triethoxycaprylylsilane), a coating agent commonly used in sunscreens have lesser photocatalytic activities than do uncoated ZnO-NPs^21^. Uncoated ZnO-NPs induced antioxidant defense mechanisms and reduced viability of human liver stellate cells^22^. However, treatment with two coated ZnO-NPs (HP1 and MAX; dimethoxydiphenylsilane and triethoxycaprylylsilane crosspolymer) or Zn^2+^ from ZnSO_4_ had little effect on these cells. Such differences in toxic potencies among Zn^2+^, coated and uncoated ZnO-NPs were supported by differentially-expressed genes in hepatic stellate cells^22^. The water flea, *Daphnia magna* was, however, more susceptible to ZnO-NPs coated with HP1 (48-h EC50 = 1.1 mg/L) and ZnO-NPs coated with MAX (48-h EC50 = 1.0 mg/L) than uncoated ZnO-NPs (48-h EC50 = 7.5 mg/L) in M4 medium^23^. Toxicity of ZnO-NPs coated with HP-1 to *D*. *magna* decreased significantly in natural spring water and pond water, with 48-h EC50 values of >100 and 13.4 mg/L, respectively^23^. Toxic potencies of nanoparticles were also dependent on types of cells exposed and culture media used during testing^24^.

Of the literature addressing toxic potencies of coated ZnO-NPs, little information has been published on aquatic organisms, particularly phytoplankton. In the present study, three freshwater microalgae, *Chlamydomonas reinhardtii*, *Chlorella pyrenoidosa* and *Pseudokirchneriella subcapitata*, and three marine microalgae, *Thalassiosira pseudonana*, *Thalassiosira weissflogii* and *Isochrysis galbana*, were exposed to water-borne coated and uncoated ZnO-NPs. Micron-sized ZnO and Zn^2+^ from ZnSO_4_ were also studied to determine effects of particle size and concentrations of Zn^2+^, respectively. It was hypothesized that modification of surfaces of ZnO-NPs would alter physicochemical behavior of ZnO-NPs such as aggregation and release of Zn^2+^, and hence alter toxic potencies of ZnO-NPs to various microalgae. Because coating material, which is covalently bonded onto the surface of ZnO-NPs, might hinder release of Zn^2+^ from ZnO-NPs, toxic potency of ZnO-NPs might decrease. Here, two silane-coated ZnO-NPs were tested, namely 3-aminopropyltrimethoxysilane-coated ZnO-NPs (A-ZnO-NPs) and dodecyltrichlorosilane-coated ZnO-NPs (D-ZnO-NPs). 3-Aminopropyltrimethoxysilane is an effective capping agent used to control sizes of ZnO-NPs crystallites and to stabilize ZnO-NPs in colloidal suspensions^25^. Dodecyltrichlorosilane, which shares a similar chemical structure with HP1, provides a hydrophobic surface to ZnO-NPs. The present study had three primary goals to: (1) compare the physicochemical characteristics of coated and uncoated ZnO-NPs among several culture media; (2) investigate their acute toxicities to selected freshwater and marine microalgae species, and (3) determine molecular mechanisms of toxic action in a marine diatom species.

## Results

### Mean sizes of particles

**Figure 1:**
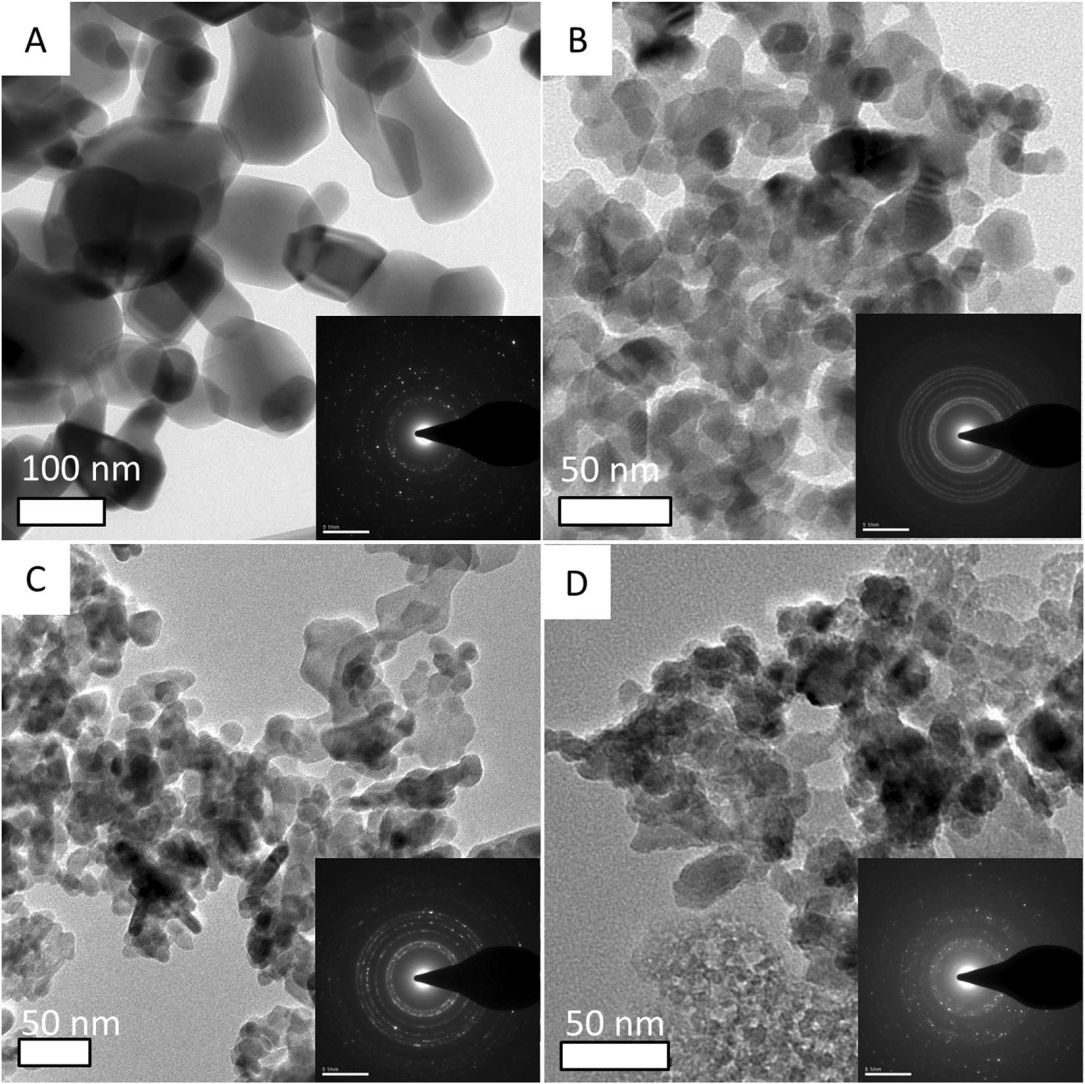
TEM images of (**A**) ZnO; (**B**) ZnO-NPs; (**C**) A-ZnO-NPs and (**D**) D-ZnO-NPs dry powder. Inset: electron diffraction pattern of the particles.

Size of particles of ZnO (121 ± 9 nm; mean ± 95% confidence interval) were significantly larger than of ZnO-NPs (23 ± 1 nm), A-ZnO-NPs (24 ± 1 nm) and D-ZnO-NPs (23 ± 1 nm) (Fig. 1; One-way ANOVA: *F*_*3*, *396*_ = 368.37, *p* < 0.001). There was no significant difference in particle size between the uncoated ZnO-NPs and the two coated ZnO-NPs (One-way ANOVA: *F*_*2*, *297*_ = 0.838, *p* > 0.05). The coating materials on the coated ZnO-NPs were not obviously observed by TEM since they were not in crystalline structure.

## Aggregation

**Figure 2:**
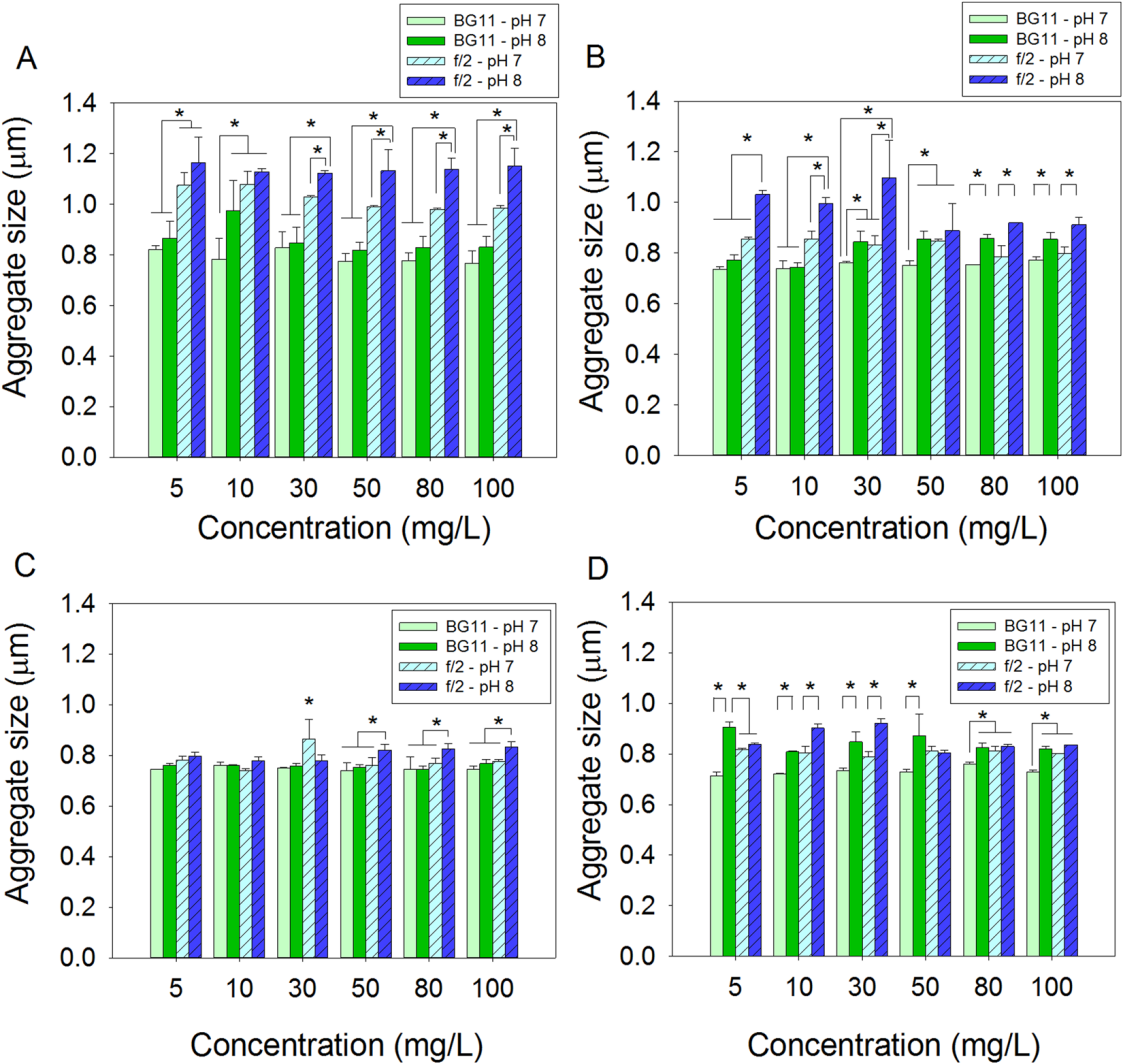
Mean aggregation size of: (**A**) ZnO; (B) ZnO-NPs; (**C**) A-ZnO-NPs and (**D**) D-ZnO-NPs after seven days of exposure at 25 °C (mean and 95% confidence interval, *n* = 3). *Denoted significant different among different treatments within each exposure concentration (one-way ANOVA, followed by post-hoc Tukey’s test, *p* < 0.05).

Micron-sized ZnO, ZnO-NPs and the two coated ZnO-NPs aggregated in both BG-11 and f/2 algal culture media at pH 7 and pH 8 (Fig. 2). Types of particles (ZnO, ZnO-NPs, A-ZnO-NPs and D-ZnO-NPs), concentration, culture medium and pH of the medium all affected sizes of aggregation (Four-way ANOVA: *F*_*15*, *192*_ = 5.27, *p* < 0.001).

In general, micron-sized ZnO formed larger aggregates than the nanoparticles (post-hoc Tukey’s test, *p* < 0.05). Among the nanoparticles, uncoated ZnO-NPs formed larger aggregates than D-ZnO-NPs, followed by A-ZnO-NPs (post-hoc Tukey’s test, *p* < 0.05). The effect of pH on the aggregation size of the test particles was dependent on the medium. In freshwater-based BG-11 medium, the size of aggregation of ZnO-NPs and D-ZnO-NPs was significantly increased from pH 7 to pH 8 (Two-way ANOVA: ZnO-NPs: *F*_*1*,*24*_ = 109.86; *p* < 0.001; D-ZnO-NPs: *F*_*1*,*24*_ = 179.21, *p* < 0.001). However, the effect of pH on the aggregation size of ZnO and A-ZnO-NPs in BG-11 medium was not significant (Two-way ANOVA: ZnO: *F*_*1*,*24*_ = 2.28, *p* > 0.05; A-ZnO-NPs: *F*_*1*,*24*_ = 3.15, *p* > 0.05). In seawater-based f/2 medium, the size of aggregation of the four particles increased from pH 7 to pH 8. Aggregation was generally enhanced in f/2 medium when compared to BG-11 medium.

## Dissolution

**Figure 3:**
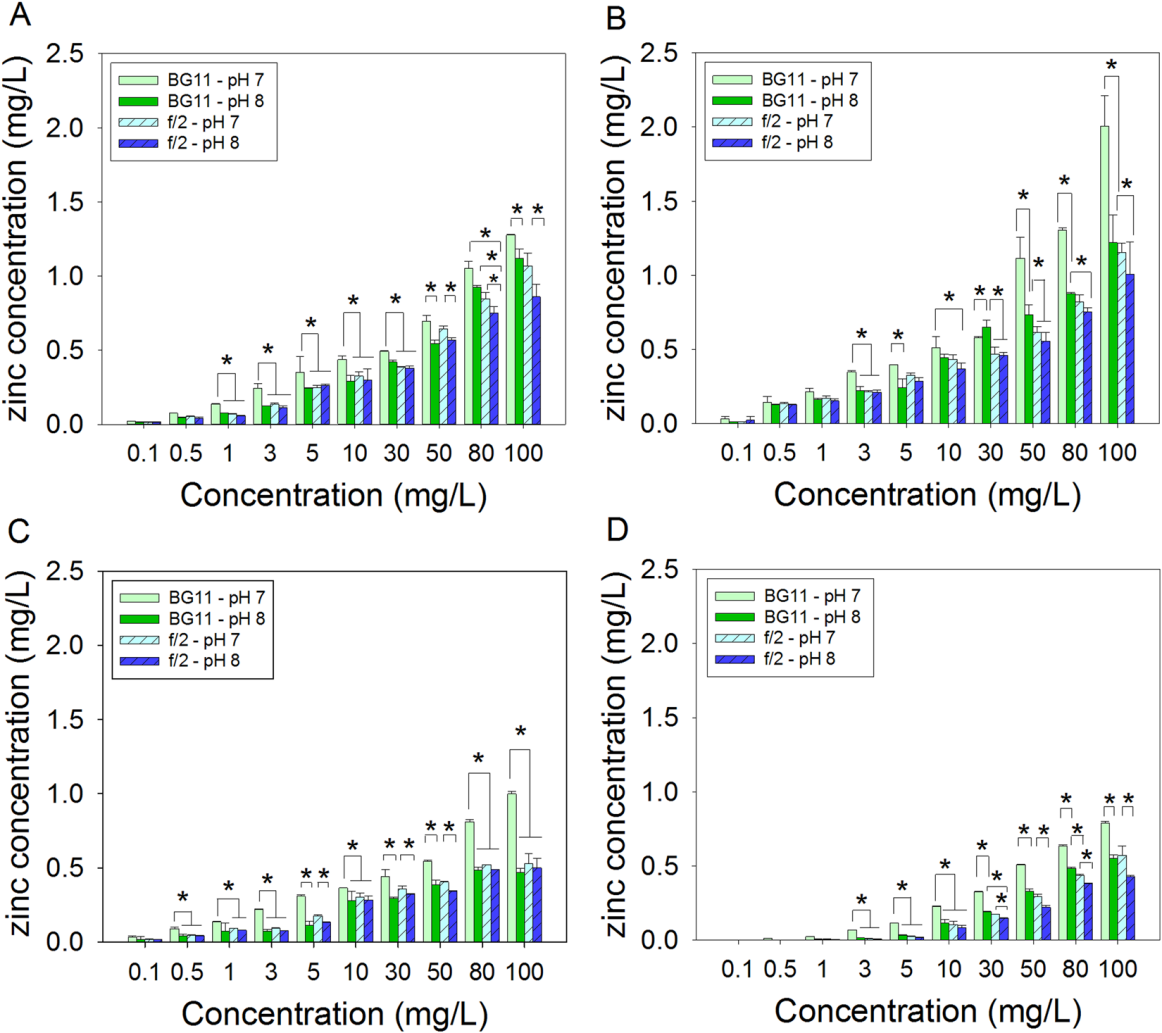
Mean concentrations of dissolved zinc in suspensions of: (**A**) ZnO; (B) ZnO-NPs; (**C**) A-ZnO-NPs and (**D**) D-ZnO-NPs at various concentrations after seven days of exposure at 25 °C (mean and 95% confidence interval, *n* = 3). *Denoted significant different among different treatments within each exposure concentration (one-way ANOVA, followed by post-hoc Tukey’s test, *p < *0.05).

Types of particles, concentration, culture media and pH of the media exhibited interactions on dissolutions of the four test particles (i.e., ZnO, ZnO-NPs, A-ZnO-NPs and D-ZnO-NPs) (Four-way ANOVA: *F*_*27*, *320*_ = 8.32, *p* < 0.001) (Fig. 3). Dissolutions of particles followed the order: ZnO-NPs > ZnO > A-ZnO-NPs > D-ZnO-NPs in both culture media at pH 7 and 8 (post-hoc Tukey’s test: *p* < 0.05). Particles dissolved better at pH 7 than at pH 8, and BG-11 medium was a better solvent than was f/2 medium.

### 96-h inhibition of growth of algae

**Figure 4:**
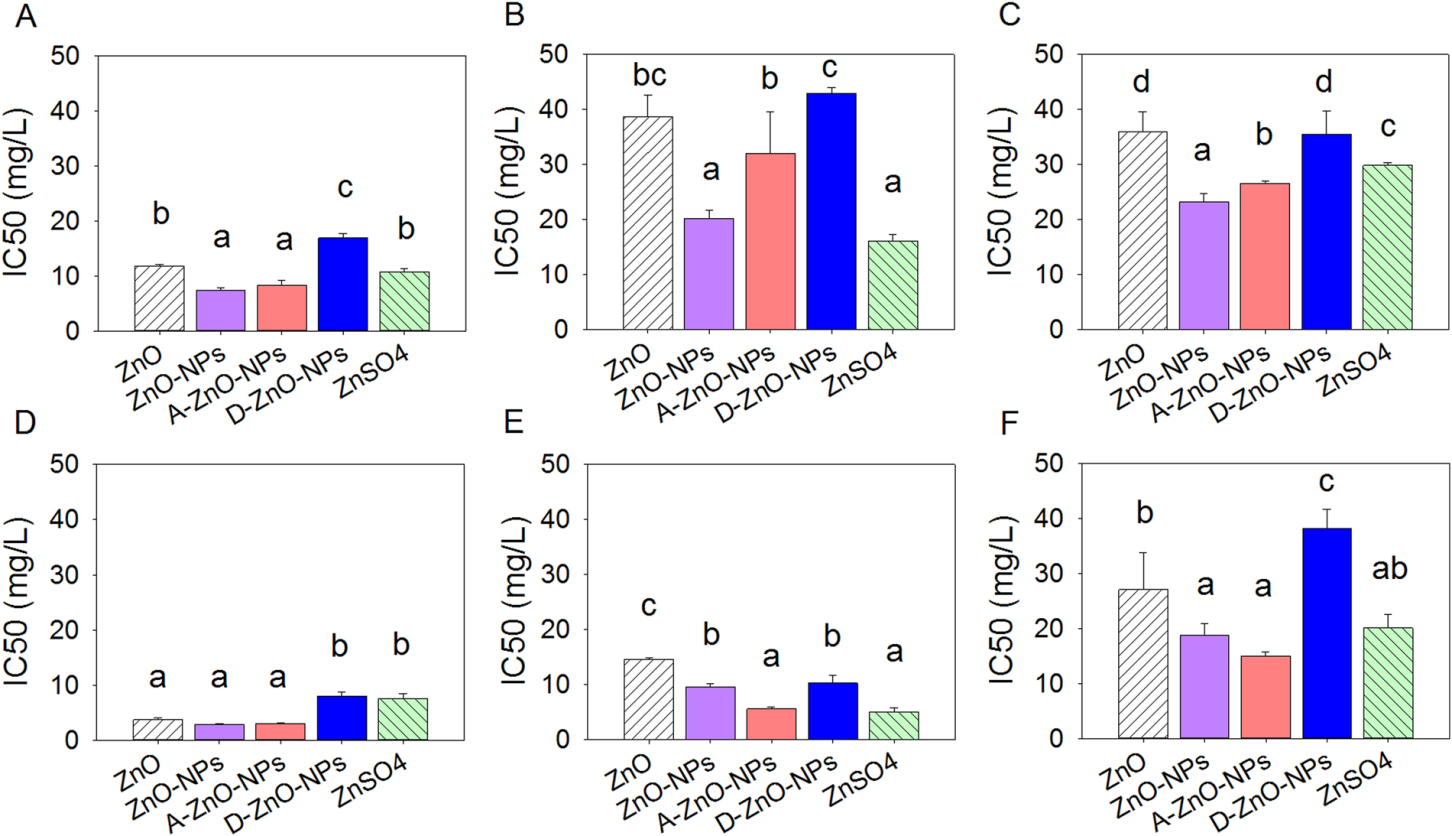
96-hour median inhibition concentration (IC50) based on growth inhibition of the five test chemicals: ZnO, ZnO-NPs, A-ZnO-NPs, D-ZnO-NPs and ZnSO_4_ to the freshwater microalgae (**A**) *Chlamydomonas reinhardtii*; (**B**) *Chlorella pyrenoidosa* and (**C**) *Pseudokirchneriella subcapitata* and the marine microalgae (**D**) *Thalassiosira pseudonana*; (**E**) *Thalassiosira weissflogii* and (**F**) *Isochrysis galbana* (mean and 95% confidence interval, *n* = 4). Bars with different letters denoted that the IC50s were different significantly between chemicals (one-way ANOVA, followed by post-hoc Tukey’s test, *p* < 0.05).

**Figure 5:**
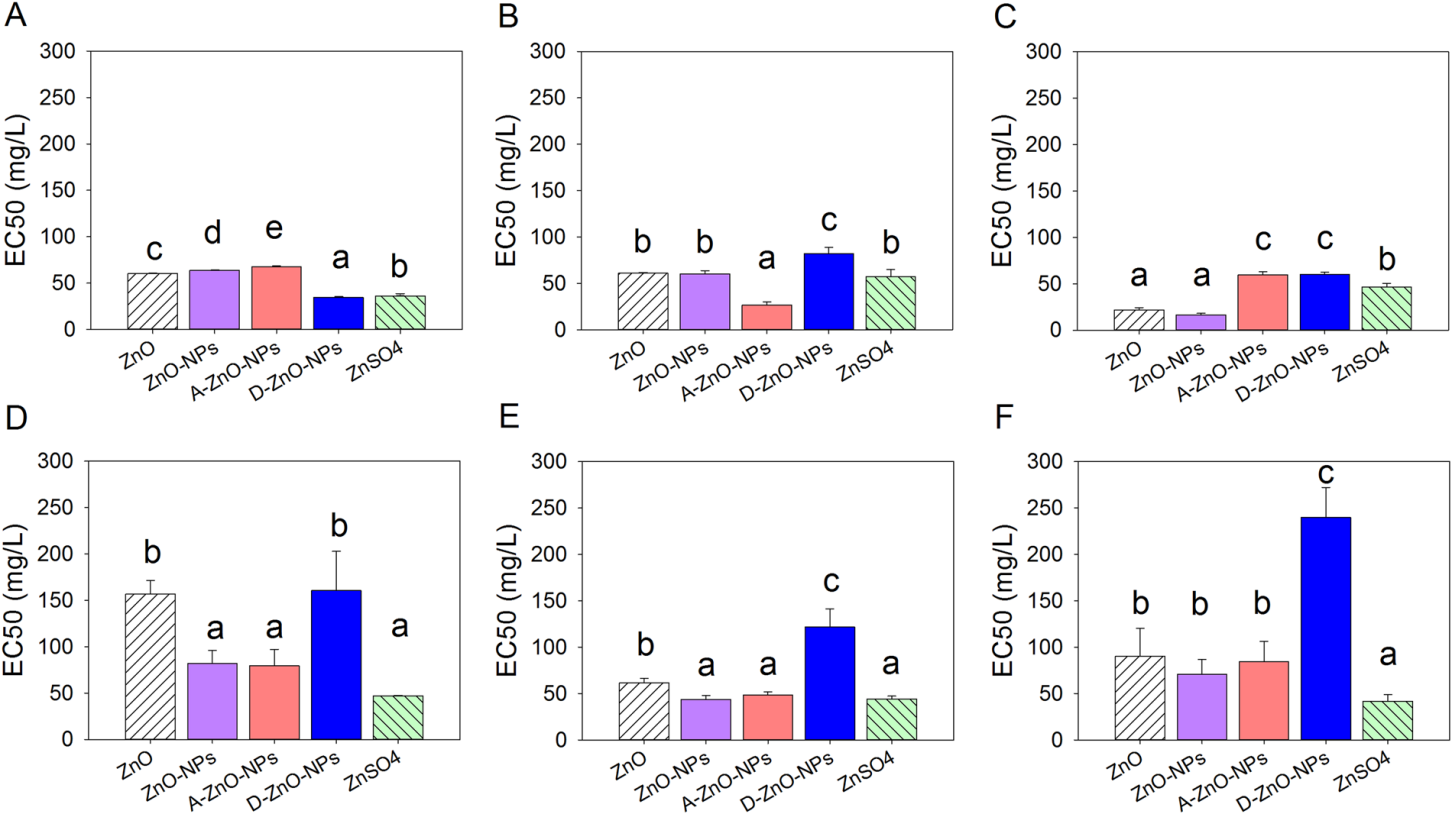
96-hour median effect concentration (EC50) on photosynthesis response in terms of the relative Ф_Po_ of the five test chemicals: ZnO, ZnO-NPs, A-ZnO-NPs, D-ZnO-NPs and ZnSO_4_ to the freshwater microalgae (**A**) *Chlamydomonas reinhardtii*; (**B**) *Chlorella pyrenoidosa* and (**C**) *Pseudokirchneriella subcapitata* and the marine microalgae (**D**) *Thalassiosira pseudonana*; (**E**) *Thalassiosira weissflogii* and (**F**) *Isochrysis galbana* (mean and 95% confidence interval, *n* = 4). Bars with different letters denoted that the EC50s were different significantly between chemicals (one-way ANOVA, followed by post-hoc Tukey’s test, *p* < 0.05).

**Figure 6:**
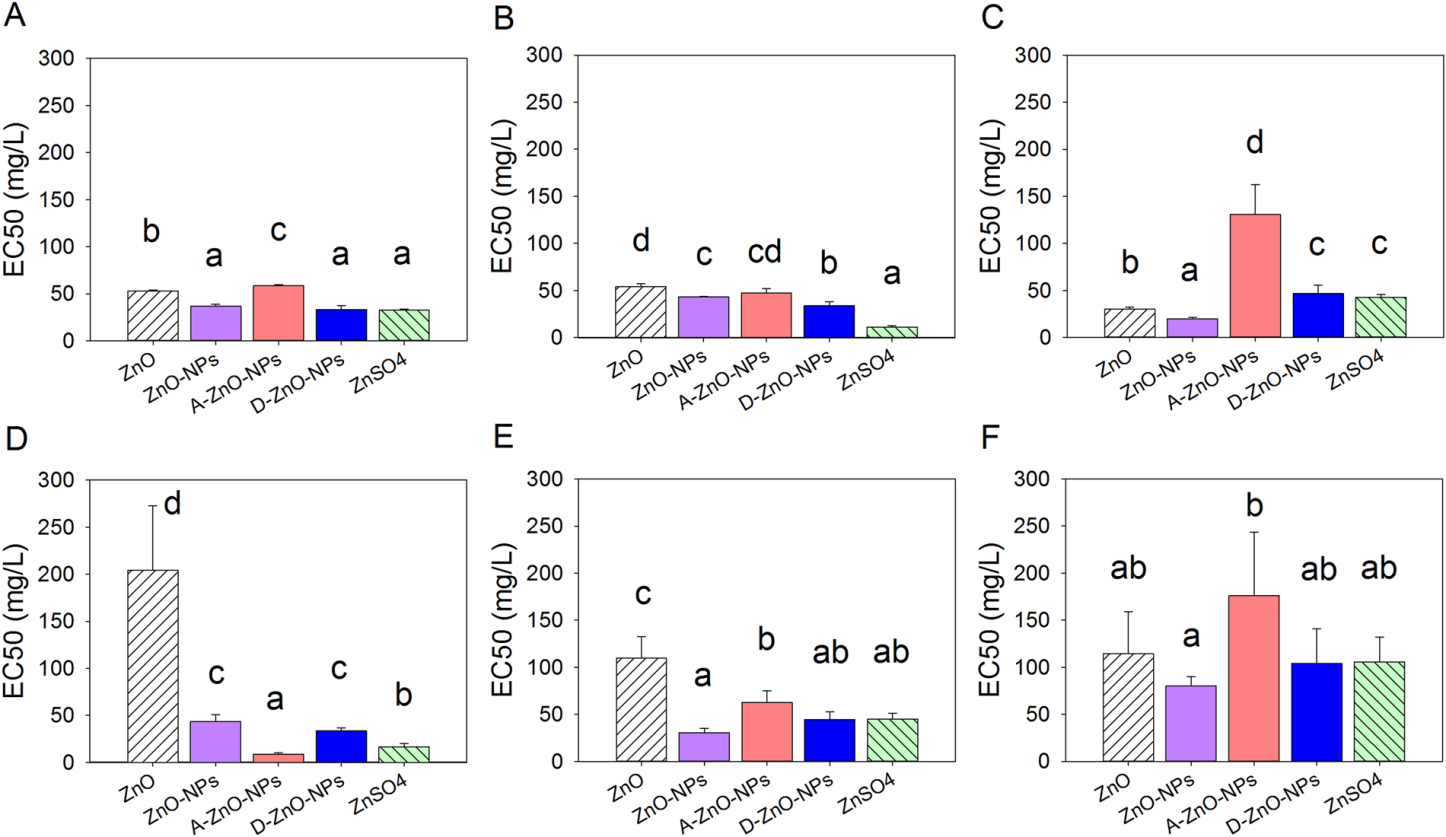
96-hour median effect concentration (EC50) on photosynthesis response in terms of the relative Ф_2_ of the five test chemicals: ZnO, ZnO-NPs, A-ZnO-NPs, D-ZnO-NPs and ZnSO_4_ to the freshwater microalgae (**A**) *Chlamydomonas reinhardtii*; (**B**) *Chlorella pyrenoidosa* and (**C**) *Pseudokirchneriella subcapitata* and the marine microalgae (**D**) *Thalassiosira pseudonana*; (**E**) *Thalassiosira weissflogii* and (**F**) *Isochrysis galbana* (mean and 95% confidence interval, *n* = 4). Bars with different letters denoted that the EC50s were different significantly between chemicals (one-way ANOVA, followed by post-hoc Tukey’s test, *p* < 0.05).

Based on comparisons of IC50 or EC50 values, the toxicities of the five test zinc compounds to freshwater and marine microalgae were species-dependent (Figs 4, 5 and 6). Both chemicals and species of alga had effects on IC50 and EC50 values (Two-way ANOVA: growth inhibition: *F*_*20*, *90*_ = 15.48; relative Ф_Po_: *F*_*20*, *90*_ = 24.16; relative Ф_2_: *F*_*20*, *90*_ = 11.45, all *p* values < 0.001). Based on growth inhibition and photosynthesis as measured by maximum quantum yield of photosystem II photochemistry (i.e., relative Ф_Po_), ZnO-NPs, A-ZnO-NPs and ZnSO_4_ were more toxic to the microalgae than ZnO, followed by D-ZnO-NPs (Figs 4 and 5) (post-hoc Tukey’s test, *p* < 0.05). However, based on photosynthesis response as measured by effective quantum yield of chemical energy conversion in photosystem II (i.e., relative Ф_2_), ZnO-NPs, D-ZnO-NPs and ZnSO_4_ were more toxic to the microalgae than A-ZnO-NPs, followed by ZnO (Fig. 6) (post-hoc Tukey’s test, *p* < 0.05).

Based on multivariate analysis of growth by comparing their IC50 values, *T*. *pseudonana*, *T*. *weissflogii* and *C*. *reinhardtii* were more sensitive to ZnO-NPs, A-ZnO-NPs and ZnSO_4_ (SI, Fig. S3). The freshwater algae *C*. *reinhardtii*, *C*. *pyrenoidosa* and *P*. *subcapitata* were more sensitive than the marine algae *T*. *pseudonana* and *I*. *galbana* in terms of photosynthesis inhibition by comparing EC50 values (SI, Fig. S3).

### Morphology of microalgae analyzed by scanning electron microscopy (SEM)

**Figure 7:**
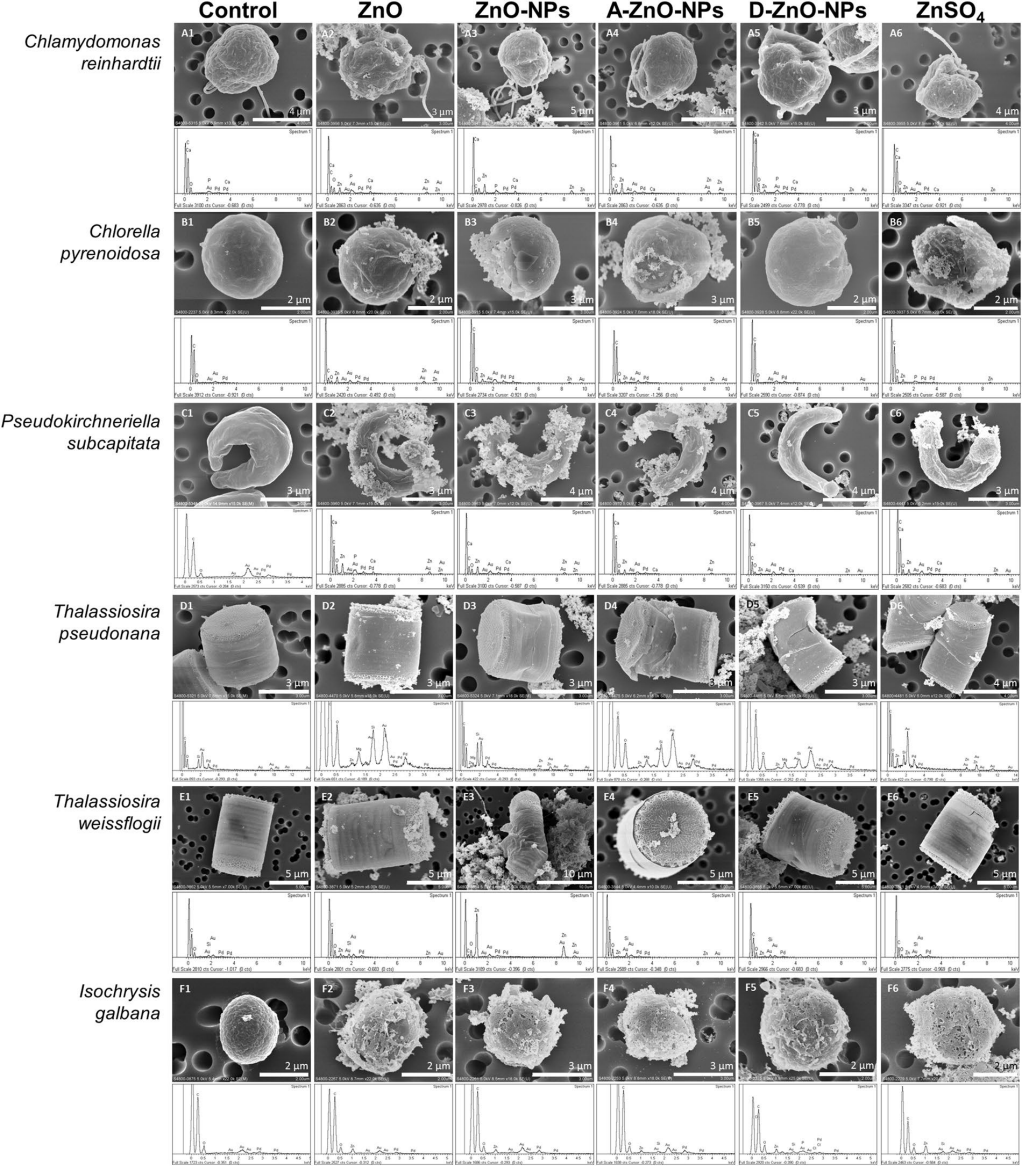
SEM images of the microalgae. (A1-A6): *Chlamydomonas reinhardtii*; (B1-B6): *Chlorella pyrenoidosa*; (C1-C6): *Pseudokirchneriella subcapitata*; (D1-D6): *Thalassiosira pseudonana*; (E1-E6): *Thalassiosira weissflogii*; (F1-F6): *Isochrysis galbana*. The microalgae were exposed to the control and 10 mg/L of each of the five test chemicals for 4 days. The spectrum below each SEM image showed the elemental composition of the detected elements by energy dispersive spectroscopy. High Au and Pd peaks in energy dispersive spectroscopy (EDS) spectrum were due to Au/Pd sputter coating on the sample surface.

The microalgae in the control group had normal cell structure with intact cell surface (Fig. 7: A1–F1). However the microalgae exposed to 10 mg/L of test chemicals (i.e., ZnO, ZnO-NPs, A-ZnO-NPs, D-ZnO-NPs and ZnSO_4_) showed irregular cell outlines and damaged cell surfaces (Fig. 7: A2–A6; B2-B6; C2-C6; D2-D6; E2-E6 and F2-F6). Further analysis was done by energy dispersive spectroscopy (EDS) to analyze the elemental composition on cell surface, and confirmed the presence of Zn on the microalgae which were exposed to the test chemicals (Fig. 7). No Zn was found on the surface of the control microalgae.

## Differentially Expressed Genes

**Figure 8:**
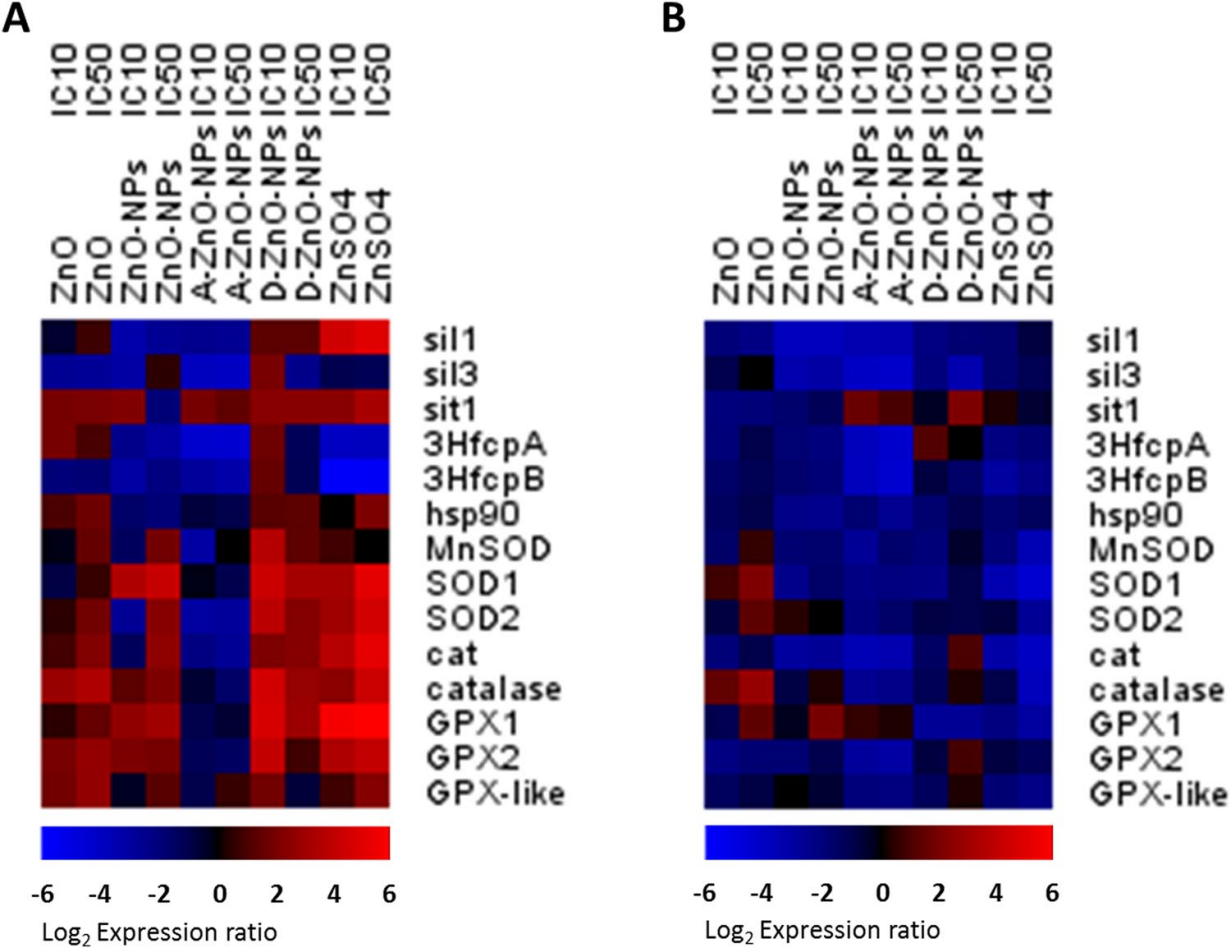
Heatmaps of expressed genes in *T*. *pseudonana* upon exposure to ZnO, ZnO-NPs, A-ZnO-NPs, D-ZnO-NPs and ZnSO_4_ at (**A**) 48 h and (**B**) 96 h. The *Gapdh* gene was used as a reference housekeeping gene to normalize the expression.

Patterns of expressions of genes were time-dependent and the five test compounds containing Zn caused different patterns of expression of genes in *T*. *pseudonana*. This indicated dissimilar mechanisms of toxic actions, especially during the first 48 h (SI, Fig. S4). The fixed factors: chemicals, exposure concentration and time affected regulation of genes in *T*. *pseudonana* (PERMANOVA: *pseudo* F = 14.64, *p* < 0.01) (SI, Fig. S4). The five test chemicals generated different patterns of expressions of genes in *T*. *pseudonana* (ANOSIM: *global* R = 0.18, *p* < 0.01). The *sil1*, *sil3* genes, which are involved in formation of silica frustules, were down-regulated by exposure to ZnO, ZnO-NPs and A-ZnO-NPs for both 48 and 96 h. The *3HfcpA* and *3HfcpB* genes, which are involved in photosynthetic activity, were generally down-regulated by ZnO-NPs, A-ZnO-NPs and ZnSO_4_ after both 48 and 96 h of exposure. *SOD*, *cat* and *GPX*, which are related to oxidative stress, were mostly up-regulated by exposure to ZnO, ZnO-NPs, D-ZnO-NPs and ZnSO_4_ after 48 h, and then down-regulated after 96 h (Fig. 8). Main effects of duration and magnitude of exposure interacted on regulations of genes (time: ANOSIM *global* R = 0.16, *p* < 0.01; concentration: ANOSIM *global* R = 0.22, *p* < 0.01). However, effects of exposure concentrations of the chemicals (i.e., IC10 and IC50) were not significantly different (*p* > 0.05).

## Discussion

Types of exposure media, pH, size of the particles and the coating materials affected aggregation and dissolution of particles. The seawater-based f/2 medium enhanced aggregation of the four types of particles, compared to freshwater-based BG-11 medium. This might have been due to greater ionic strength of seawater compressing the electric double layer of particles and reduced repulsive forces between particles, which facilitated aggregation^26^. Similar phenomena have been previously reported^27–29^. TiO_2_, CeO_2_ and ZnO nanoparticles formed larger aggregates in higher ionic strength seawater, while these three metal oxide nanoparticles formed smaller aggregations in freshwater medium^28^. pH of the medium also affected size of aggregation. Sizes of aggregation of the four test particles were proportional to pH from pH 7 to pH 8. This might have been due to pH 8 being closer to the reported isoelectric point of ZnO, which ranged from pH 8.7 to 10.3^30^, at which the electrostatic repulsive interactions between ZnO-NPs decreased, and aggregation was enhanced^26^. These results were consistent with those reported previously^26^ that rate of sedimentation of ZnO-NPs was greater at pH 9 than at pH 7. However, pH did not significantly affect the zeta potential of ZnO, ZnO-NPs and two coated ZnO-NPs (SI, Fig S1). Since the four Zn-containing particles had mean zeta potential within ±30 mV in both BG-11 and f/2 algal culture media at pH 7 and 8, they would aggregate readily in both culture media at both pH 7 and 8.

Micron-sized ZnO formed larger aggregates because of its significantly larger particle size, when compared to coated or uncoated ZnO-NPs (Fig. 1). Coating of nanoparticles also influenced aggregation of ZnO-NPs. In f/2 medium, ZnO-NPs formed larger aggregates than did D-ZnO-NPs, followed by A-ZnO-NPs. The coating materials were designed to prevent aggregation of ZnO-NPs. The organic chains of the coating materials on the ZnO-NPs surface build steric hindrance between the nanoparticles, decrease the surface energy of ZnO-NPs and prevent aggregation of A-ZnO-NPs and D-ZnO-NPs when compared to uncoated ZnO-NPs^31^. The coating materials were covalently bonded to the surface of nanoparticles and the surface chemistry of the two coated ZnO-NPs and uncoated ZnO-NPs was determined by use of FT-IR spectroscopy (SI, Fig. S2).

In this study, more dissolved zinc was measured in BG-11 medium than in f/2 medium. The greater ionic strength in f/2 medium enhanced complexation of Zn^2+^, thereby resulted in lesser concentrations of soluble Zn in the medium^32^. Increasing pH from 7 to 8 also reduced dissolution of the four Zn-containing compounds. In deionized water of ionic strength 0.1 mM NaCl, solubility of ZnO-NPs decreased from 40% at pH 7.6 to 2.4% at pH 8.3^33^. In synthetic seawater, solubility of ZnO-NPs was inversely proportional to pH, decreasing from 3.3% at pH 7.7 to 1.0% at pH 8.2^34^. Higher pH (>pH 7) is associated with greater concentrations of hydroxide (OH^−^) ions in the medium, which resulted in enhanced formation of zinc hydroxide, which in turn resulted in lesser concentrations of free Zn^2+^ ions in the medium^30,33^. In the present study, uncoated ZnO-NPs and ZnO dissolved better than the two coated ZnO-NPs. The coating materials hindered release of Zn^2+^ from the core particles. These results are consistent with those of previous studies^35^ that demonstrated that in soil pore water ZnO-NPs coated with triethoxyoctylsilane were less soluble than uncoated ZnO-NPs. In summary, concentrations of dissolved Zn in BG-11 medium at pH 7 was greater compared to f/2 medium at pH 8, and uncoated ZnO-NPs were more soluble than the two coated ZnO-NPs.

Surface modification of ZnO-NPs altered toxicities of ZnO-NPs to the studied freshwater and marine microalgae. Based on inhibition of growth, D-ZnO-NPs exerted less toxic potencies than A-ZnO-NPs and uncoated ZnO-NPs towards freshwater algae (*C*. *reinhardtii*, *C*. *pyrenoidosa*, and *P*. *subcapitata*) and marine microalgae (*T*. *pseudonana* and *I*. *galbana*). The potencies of uncoated ZnO-NPs and A-ZnO-NPs were probably attributed to their release of Zn^2+^ that caused formation of ROS.

A-ZnO-NPs and uncoated ZnO-NPs have been reported to have similar antibacterial activity to the bacterium *Bacillus atrophaeus* although A-ZnO-NPs generated lesser amounts of ROS than did uncoated ZnO-NPs, while D-ZnO-NPs exhibited the least antibacterial effect on *B*. *atrophaeus* ^36^. The coating material of A-ZnO-NPs with an amino functional group could interact with cell proteins of bacteria and damage cell membranes by changing their permeability^36^. Uncoated and aminopropyl-triethoxysilane-coated superparamagnetic iron oxide nanoparticles had similar antibacterial effects toward bacteria *Staphylococcus epidermidis* and *Staphylococcus aureus* ^37^, which suggested that antibacterial activity was related to oxidative stress and interactions between nanoparticles and bacterial cell membranes or cell proteins, resulted in physical damage and bacterial cell death. Lesser toxic potency of D-ZnO-NPs can be explained by the possibility that use of that coating could prevent release of ROS from the ZnO-NP core to the surrounding environment, and reduce photocatalytic activities of nanoparticles, and thus lower their toxicity to organisms^21^. In terms of relative Ф_2_ (i.e., the effective quantum yield of chemical energy conversion), ZnO-NPs and D-ZnO-NPs had greater toxic potencies than did A-ZnO-NPs, which implied that D-ZnO-NPs would disturb photosynthetic processes of microalgae although the effect may not be lethal and not result in inhibition of growth of microalgae. As observed from the SEM images (Fig. 7), the cell surfaces of microalgae were damaged by both coated and uncoated nanoparticles. As aggregates of nanoparticles were found on the cell surface, the interaction of the nanoparticles and the algal cells might cause malformation of the algae and inhibit their growth.

Toxic potencies of uncoated and two of the coated ZnO-NPs were species-specific. Opposite to effects on marine algae, although more Zn^2+^ ions were available in freshwater BG-11 medium, not all species of freshwater algae were more susceptible to effects of uncoated or coated ZnO-NPs. The marine diatoms *T*. *pseudonana* and *T*. *weissflogii*, and freshwater alga *C*. *reinhardtii* were more sensitive to ZnO-NPs, A-ZnO-NPs and ZnSO_4_, implying that these three algal species were more susceptible to Zn^2+^ and ROS. Both marine diatoms *T*. *pseudonana* and *T*. *weissflogii* containing silica frustules were susceptible to exposure to ZnSO_4_ because excess uptake of Zn^2+^ would compete for uptake of silica by algal cells and consequently impair formation of frustules and growth^38^. The freshwater alga *C*. *reinhardtii* was more sensitive to ZnO-NPs and Zn^2+^ than were other freshwater algae, possibly because it is known to have relatively great fluxes of uptake of metal ions^39^. *C*. *reinhardtii* has been reported to be more sensitive to silver (Ag) than *P*. *subcapitata*, because *C*. *reinhardtii* had a fast silver uptake rate such that it absorbed all dissolved silver ions species; while *P*. *subcapitata* with a slow silver uptake rate, it absorbed mainly free silver ions^39^. These two freshwater algae had different target sites for uptake of Ag. Intracellular targets for *C*. *reinhardtii* were probably proteins and enzymes, whereas the photosynthetic apparatus was the intracellular target in *P*. *subcapitata* ^40^. Based on inhibition of growth, the freshwater alga *C*. *pyrenoidosa* was less sensitive to ZnO-NPs and Zn^2+^ than was *C*. *reinhardtii*, possibly because species of the genus *Chlorella* could regulate internal concentration of Zn. An increase in concentration of Zn to which *Chlorella* sp. were exposed would not increase the intracellular concentration of Zn^41^.

Profiles of expressions of genes for *T*. *pseudonana* suggested distinguishable toxic mechanisms between ZnO-NPs and Zn^2+^. Similar findings have also been reported by previous studies in human hepatic cells^22^ and bacterial cells^42^. In the study, results of which are presented here, when *T*. *pseudonana* was exposed to ZnO-NPs and ZnSO_4_, the diatoms were under oxidative stress, as indicated by the up-regulation of the *SOD* genes (*SOD1* and *SOD2*), the cat genes (*cat* and *catalase*) and the *GPX* genes (*GPX1* and *GPX2*)^43–45^, particularly when exposed to greater concentrations (IC50 of ZnO-NPs: 0.21 mg/L of Zn; IC50 of ZnSO_4_: 1.15 mg/L of Zn) in 48 h. Evidently, both ZnO-NPs and associated Zn^2+^ induced oxidative stress in *T*. *pseudonana*. Such results are consistent with those reported previously that both ZnO-NPs and dissolved zinc can induce oxidative stress in human immune cells^46^. Moreover, oxidative stress induced by ZnSO_4_ in *T*. *pseudonana* was more prominent, which indicated that up-regulation of *SOD*, *cat* and *GPX*, possibly due to a greater concentration of dissolved Zn^2+^ was released from ZnSO_4_ compared to ZnO-NPs during the gene expression experiment. However, as observed for inhibition of growth of *T*. *pseudonana* (Fig. 4D), ZnO-NPs were more toxic than ZnSO_4_. These results suggest that the greater amount of oxidative stress induced in *T*. *pseudonana* by Zn^2+^ might not be the sole factor in governing inhibition of growth.

**Figure 9:**
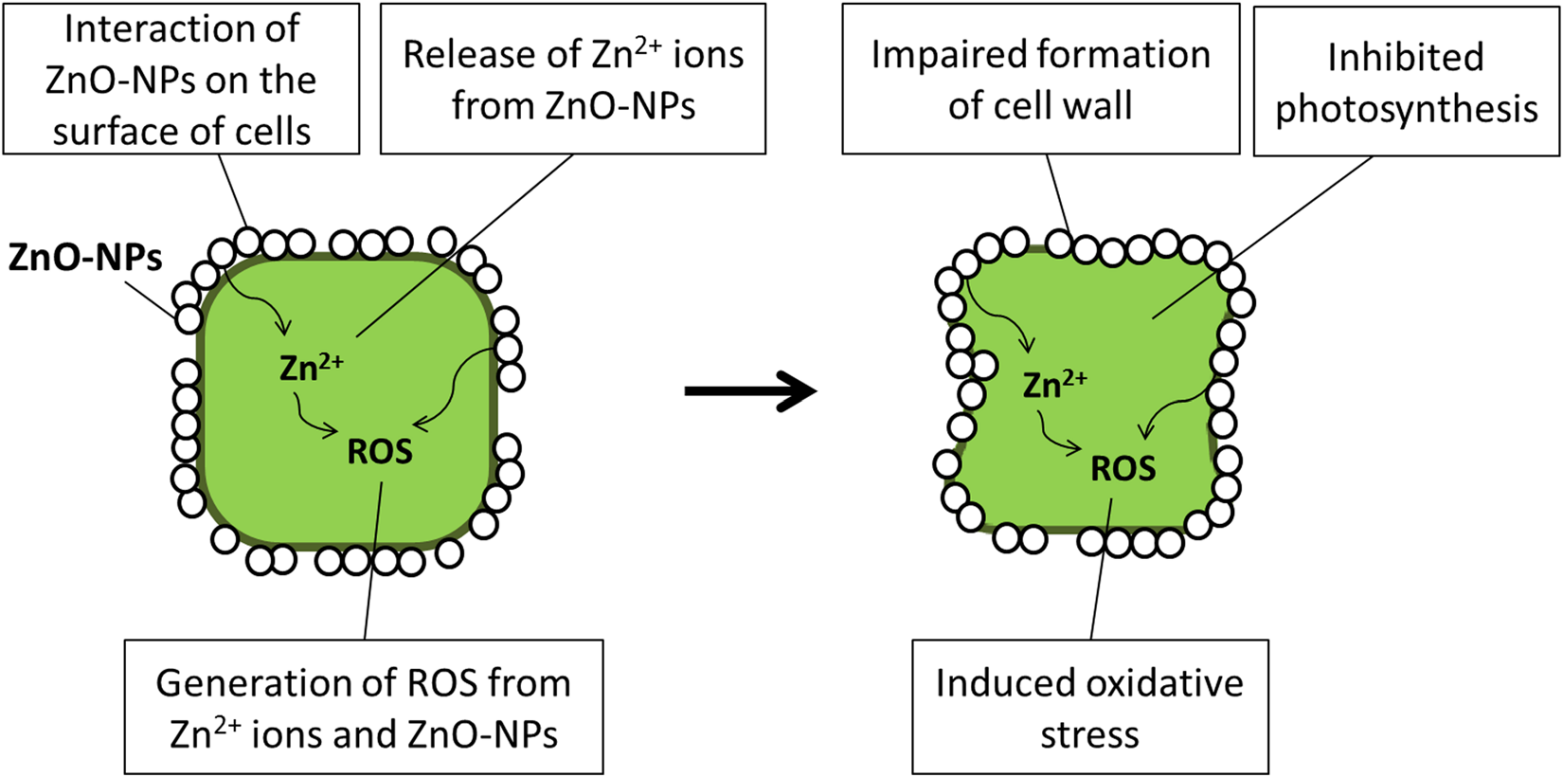
A schematic diagram of the proposed mechanism of the interaction of ZnO-NPs and the algal cell of *T*. *pseudonana*.

ZnO-NPs impaired formation of silica frustules and transport of silicon into diatoms during the first 48 h even at small concentrations (IC10: 0.18 mg/L of Zn) (Fig. 8). This proposed mechanism was supported by down-regulation of *sil1*, *sil3* and *sit1* genes^47,48^. Alternatively, up-regulations of *sil1* and *sit1* were observed during the first 48 h when exposed to ZnSO_4_ exposure at the first 48 h. No significant regulations of the *sil1*, *sil3* and *sit1* were observed at 96 h, which suggested that ZnSO_4_ would have less effect on formation of silica frustules of diatoms than did ZnO-NPs. Also, down-regulation of *3HfcpA* and *3HfcpB* by ZnO-NPs and ZnSO_4_ indicated that photosynthetic activity of the diatom was disturbed^47^. *T*. *pseudonana* exposed to micron-sized ZnO exhibited similar patterns of expression of genes as did those exposed to ZnO-NPs, except for the *3HfcpA* gene, which was slightly up-regulated by ZnO. This result implies that ZnO would have less damaging effects on photosynthetic activity of this species of diatom. On the basis of our findings and observations (Figs 7 and 8), we propose a probable mechanism of the interaction of ZnO-NPs and the marine diatom *T*. *pseudonana* (Fig. 9). The introduced ZnO-NPs can impair the formation of silica frustule of the diatom. Both the dissolved Zn^2+^ ions and the nanoparticles can disturb and inhibit the photosynthesis activity, and induce oxidative stress in the algal cell. As a result, the growth of the diatom was inhibited. This proposed toxicity mechanism of ZnO-NPs was also supported by previous studies^49–51^.

Modulation of surfaces of ZnO-NPs significantly influenced mechanisms of toxic action of ZnO-NPs to *T*. *pseudonana*. A-ZnO-NPs induced a unique pattern of expression of genes in *T*. *pseudonana* when compared to the four other Zn-containing compounds. Down-regulation of *sil1* and *sil3* indicated that A-ZnO-NPs impaired formation of silicon frustules in the diatom even when exposed to small concentrations (IC10: 0.08 mg/L of Zn)^47,48^. Down-regulation of *sil1* and *sil3* was also observed when diatoms were exposed to ZnO or ZnO-NPs. This result indicated that ZnO, uncoated ZnO-NPs and A-ZnO-NPs inhibited growth hindering formation of frustules. Down-regulations of *3HfcpA* and *3HfcpB* were also observed in diatoms exposed to A-ZnO-NPs, which indicated that A-ZnO-NPs would also decrease photosynthetic activities of diatoms^47^. However, among the five zinc-containing materials, only A-ZnO-NPs resulted in down-regulation of genes encoding for enzymes which defend against oxidative stress (i.e., *SOD*, *cat* and *GPX* genes) after 48 and 96 h of exposure. This might indicate A-ZnO-NPs did not induce oxidative stress to the diatom, but this suggestion was not consistent with previous experimental results that A-ZnO-NPs generated ROS^36^. Another possible reason is that experimental time points (48 h and 96 h) might not capture oxidative responses of *T*. *pseudonana* to A-ZnO-NPs since there might be early onset of oxidative stress genes within the first 12–24 hours of exposure and the gene expression patterns of the cells are time-dependent^52^. That is, A-ZnO-NPs might have induced oxidative stress in the diatom earlier than 48 h. When *T*. *pseudonana* was exposed to D-ZnO-NPs, formation of frustules, as indicated by down-regulation of *sil1* and *sil3* genes, was affected only after 96 h. Up-regulation of genes related to responses to oxidative stress by the diatom suggested that D-ZnO-NPs could induce oxidative stress in *T*. *pseudonana* although D-ZnO-NPs released less ROS than uncoated ZnO-NPs and A-ZnO-NPs^36^.

Coatings on surfaces of ZnO-NPs can alter physicochemical properties, and hence modify the toxic potencies of ZnO-NPs to the microalgae. As showed in the current results, based on inhibition of growth of algae, D-ZnO-NPs were less toxic than uncoated ZnO-NPs, whereas A-ZnO-NPs exhibited comparable toxic potencies as uncoated particles. These results suggest that use of D-ZnO-NPs in sunscreens might result in less adverse effects on primary producers in aquatic ecosystems.

## Conclusions

As shown by the results of this study, exposure media, pH of the media, size of the particles and coating materials all affected the aggregation and dissolution of ZnO, ZnO-NPs as well as the two silane-coated ZnO-NPs. Seawater-based f/2 medium at higher pH (i.e. pH 8) facilitated aggregation of ZnO, ZnO-NPs and the two coated ZnO-NPs; whereas the freshwater-based BG-11 medium at lower pH (i.e. pH 7) enhanced dissolution of the four test zinc compounds after seven days of exposure. Uncoated ZnO-NPs generally formed larger aggregates, but they were more soluble than the two coated ZnO-NPs, possibly due to the fact that the organic coating materials provide steric repulsion between the nanoparticles and separate the aggregations/agglomerate, and the coating materials also hindered the dissolution of the ZnO-NPs. Modification of surfaces of ZnO-NPs changed their toxic potencies to microalgae, with uncoated ZnO-NPs and A-ZnO-NPs being generally more potent at inhibiting growth and maximum quantum yield of photosystem II than D-ZnO-NPs. However, D-ZnO-NPs and uncoated ZnO-NPs were more potent at decreasing effective quantum yield of chemical energy conversion in photosystem II of microalgae than were A-ZnO-NPs. These results imply that D-ZnO-NPs might have less effect on growth inhibition but might induce sub-lethal effects to the microalgae by reducing their photosynthetic activity. Toxic potencies of uncoated and coated ZnO-NPs to microalgae were species-specific.

The two coated ZnO-NPs and ZnSO_4_ caused different profiles of expressions of genes in the marine diatom *T*. *pseudonana*. ZnO, ZnO-NPs and A-ZnO-NPs would impair formation of silica frustules and uptake of silicon by the diatom. However, ZnSO_4_ did not result in significant down-regulation of genes encoding for the frustule formation proteins. Thus, ZnSO_4_ would have less effect on formation of cell walls. ZnO, ZnO-NPs, D-ZnO-NPs and ZnSO_4_ could induce oxidative stress in *T*. *pseudonana*; while ZnO-NPs, A-ZnO-NPs and ZnSO_4_ would disturb the photosynthetic activity of the diatom. ZnO-NPs and their associated Zn^2+^ had different toxic mechanisms towards *T*. *pseudonana*; furthermore, the surface modification would alter the mechanisms of toxicity of ZnO-NPs to *T*. *pseudonana*. Since D-ZnO-NPs are generally less toxic than ZnO-NPs to the microalgae, ZnO-NPs coated with hydrophobic alkyl chains might be adopted as an active ingredient in eco-friendly sunscreens.

## Methods

### Chemical preparation

Uncoated ZnO-NPs (20 nm; 99.5% purity) were purchased as dry powders from Nanostructured & Amorphous Materials Inc. (New Mexico, USA) with specific surface area of 50 m^2^/g (manufacturer’s data). ZnO-NPs with surfaces modified with 3-aminopropyl- trimethoxysilane (A-ZnO-NPs) or dodecyltrichlorosilane (D-ZnO-NPs) were synthesized in the nanomaterial laboratory of Department of Physics, the University of Hong Kong (Leung *et al*. 2012) using the same batch of ZnO-NPs powders. Zinc oxide (ZnO) and zinc sulphate (ZnSO_4_) were purchased from Sigma-Aldrich (St. Louis, MO, USA) with a purity of 99.99% and 99.999%, respectively.

### Morphology of the particles

The size and shape of ZnO, ZnO-NPs, A-ZnO-NPs and D-ZnO-NPs in dry powders were determined using a transmission electron microscope (TEM; Tecnai G2 20S-TWIN at 200 kV, Philips, Eindhoven, The Netherlands). The four zinc oxide powders were dispersed in pure ethanol, and one drop (0.7 µL) of the solution was then placed onto an ultrathin carbon-coated copper grid. All specimens were dried at room temperature before analysis. 100 particles were measured in random fields of view of three images to calculate the mean particle size using Image J software (version 1.47, National Institutes of Health, USA).

### Physicochemical characterization

Stock suspensions of ZnO, ZnO-NPs, A-ZnO-NPs and D-ZnO-NPs of 200 mg/L were prepared in triplicate in BG-11 medium (salinity: 3 practical salinity unit (PSU)) and f/2 medium (salinity: 32 PSU), respectively, with continuous stirring for 7 days at 25 ± 1 °C and at either pH 7 or pH 8. The BG-11 culture medium for freshwater algae was obtained by adding BG-11 ingredients^53^ to autoclaved milli-Q water (18.2 MΩcm) while the f/2 culture medium for marine algae was obtained by adding f/2 ingredients^54^ to autoclaved filtered artificial seawater (0.45-µm membrane filter, Millipore, Ireland). pH of culture media was adjusted by NaOH and HCl, and checked by a pH meter equipped with a digital thermometer (Mettler-Toledo AG, Switzerland). Salinity of each test solution was checked by use of a refractometer (S/Mill-E, Atago, Japan). Ten test concentrations of 0.1, 0.5, 1, 3, 5, 10, 30, 50, 80 or 100 mg/L, that were used to determine 96-h inhibition of growth of algae and characterization of physicochemical properties, were then prepared from the stock solution by serial dilution. A factorial experimental design of 2 culture media × 2 pH × 10 concentrations was applied for physicochemical analyses for four test chemicals (ZnO, uncoated ZnO-NPs, A-ZnO-NPs and D-ZnO-NPs) in triplicate. All glassware was acid-washed, rinsed with deionized water and autoclaved before use. Temperature, salinity and pH of all test suspensions were monitored daily throughout the experiment.

### Size of Aggregation

After stirring for 7 days, sizes of aggregation of ZnO, ZnO-NPs, A-ZnO-NPs and D-ZnO-NPs suspensions in each of the 40 treatments (i.e., 2 culture media × 2 pH × 10 concentrations) was determined by use of laser diffractometer (LS 13320 Series, Beckman Coulter Inc., Fullerton, CA) in triplicate (50 mL each).

## Dissolution

Dissolved concentrations of Zn^2+^ released from suspensions of ZnO, ZnO-NPs, A-ZnO-NPs or D-ZnO-NPs in each of the 40 treatments were measured after 7-day exposure. In each treatment, an aliquant of 8 mL was withdrawn and filtered through 0.02-µm sterile syringe filters (Anotop 25, Whatman, England) to remove nanoparticles. The filtrates were digested with 2% HNO_3_ and measured in triplicate by inductively coupled plasma optical emission spectrometer (ICP-OES; ICP Optima 8300, Perkin Elmer, USA). A pure AS calibration standard of Zn^2+^ (1,000 mg/L dissolved in 2% HNO_3_), which was obtained from Perkin Elmer (Waltham, MA, USA), was used for calibration. Two blank treatments, BG-11 and f/2 medium, were used as control to evaluate the background zinc ion concentration. The limit of detection of ICP-OES for Zn is 1 µg/L.

### Microalgae culture conditions

Freshwater microalgae, *C*. *reinhardtii* (FACHB-479), *C*. *pyrenoidosa* (FACHB-9) and *P*. *subcapitata* (FACHB-271, obtained from the Freshwater Algae Culture Collection at the Institute of Hydrobiology, Wuhan, China), were cultured in the laboratory at the School of Biological Science of the University of Hong Kong in autoclaved BG-11 medium^53^ under 25 ± 1 °C, pH 7 and 14 h: 10 h light: dark photoperiod. Marine microalgae *T*. *pseudonana* (CCMP 1335), *T*. *weissflogii* (CCMP 1336) and *I*. *galbana* (CCMP 1323) were obtained from the Provasoli-Guillard National Center for Marine Algae and Microbiota, (East Boothbay, Maine, USA) were cultured in the same laboratory in autoclaved f/2 medium^54^ under 25 ± 1 °C, pH 8 and 14 h: 10 h light: dark photoperiod.

### 96-h test of inhibition of growth and photosynthesis of algae

The 96-h test of inhibition of growth of algae was conducted following the OECD guidelines^55^. Three freshwater microalgae and three marine microalgae were exposed to five materials: ZnO, ZnO-NPs, A-ZnO-NPs, D-ZnO-NPs or ZnSO_4_, in a factorial experiment design with 5 chemicals × 10 concentrations (0.1, 0.5, 1, 3, 5, 10, 30, 50, 80 and 100 mg/L) alongside a control (without addition of the test chemicals); each treatment group contained four replicates. Toxic potencies of various materials were determined simultaneously by use of the same batch of microalgae with initial algal concentration of 10^5^ cells/mL. Test vials (10 mL in volume), each containing 6 mL of the test solution, were placed randomly in an environmental chamber (Adaptis A350, Conviron, Canada), shaken regularly at 25 ± 1 °C and 14 h: 10 h light: dark photoperiod for 96 h. Rates of growth of microalgae were calculated (Equation 1)

1 $$\mu =[\mathrm{ln}(N^{\prime} )\,-\,\mathrm{ln}(N)]/t$$

where *N′* is final cell count; *N* is initial cell count; and *t* is duration of in days. After exposure for 96 h, 1 mL of algal culture was sampled from each vial for enumeration of cells in triplicate using a cell counter (Multisizer II, Coulter, Fullerton, CA, USA).

The remaining algal cultures were covered with aluminum foil for 2 h and fluorescence was measured by use of a WATER-PAM chlorophyll fluorometer (Heinz Walz GmbH, Effeltrich, Germany). Two selected parameters were used to characterize photosynthesis. These included Ф_Po_ and Ф_2_, which represent maximum quantum yield of photosystem II photochemistry and the effective quantum yield of chemical energy conversion in photosystem II, respectively, were measured. Ф_Po_ indicates the photo-inhibition caused by the test chemicals to the microalgae in a dark-adapted state; Ф_2_ indicates the efficiency of the microalgae to convert light into chemical energy under steady-state lighting conditions. Photosynthetic parameters were calculated based on the equations stated in previous studies^56–58^.

### Analysis of microalgae by SEM

Treated microalgae samples which were exposed to 10 mg/L of ZnO, ZnO-NPs, A-ZnO-NPs, D-ZnO-NPs or ZnSO_4_ for 4 days and the control samples (i.e., without addition of test chemicals) were rinsed with phosphate-buffered saline thrice and fixed in 2.5% glutaraldehyde in cacodylate buffer for 24 h at 4 °C. The samples were then washed in several changes of cacodylate buffer with 0.1 M sucrose to remove excess fixative. After washing with ethanol, the samples were concentrated onto a 0.8-µm Nucleopore polycarbonate filter (Millipore, Ireland). The samples were then dried in a critical point dryer and mounted on a SEM stub with double-sided adhesive carbon discs, and coated with a thin gold/palladium layer and analyzed by a scanning electron microscope (S-4800 FEG, Hitachi, Japan) equipped with an EDS system for elemental analysis.

### Expressions of selected Genes

Because its genome sequence was well understood, the marine diatom *T*. *pseudonana* was chosen to study its molecular responses to coated and uncoated ZnO-NPs^59^. *T*. *pseudonana* was exposed to the 5 zinc materials (ZnO, ZnO-NPs, A-ZnO-NPs, D-ZnO-NPs or ZnSO_4_) at their IC10 and IC50 at 96 h (according to the 96-h algal growth inhibition test) in parallel with the control (without addition of test chemicals) in triplicate (SI, Table S1). The protocol for determining expressions of genes has been described previously^60^, except that the volume of the test solution was 1,000 mL instead of 500 mL, and two exposure time points (i.e., 48 and 96 h) were selected. Primer sequences of the reference gene and target genes were (SI, Table S2).

### Statistical analyses

Sizes of particles of ZnO, ZnO-NPs, A-ZnO-NPs and D-ZnO-NPs (dry powders) were compared by use of one-way analysis of variance (ANOVA), followed by post-hoc Tukey’s test (SPSS version 19; SPSS Inc., Chicago). Effects of the four fixed factors, chemical, culture medium, pH and exposure concentration, and their interactions on sizes of aggregation, dissolution to release Zn^2+^ and zeta potentials of ZnO, ZnO-NPs, A-ZnO-NPs and D-ZnO-NPs were compared using four-way ANOVA, followed by post-hoc Tukey’s test. Homogeneity of variance was tested with Levene’s test. When the data did not exhibit homogeneity of variance, they were log-transformed.

Inhibition of growth and alterations of photosynthesis were evaluated using GraphPad Prism 5 (GraphPad software, Inc., San Diego). Concentrations causing 10% and median inhibition (i.e., IC10s and IC50s for growth inhibition, respectively) or the median effect concentration (i.e., EC50s for photosynthesis response in terms of Ф_Po_ and Ф_2_, respectively) of the 5 test chemicals (ZnO, ZnO-NPs, A-ZnO-NPs, D-ZnO-NPs and ZnSO_4_) were obtained from the sigmoidal log (agonist)-response curve. A two-way ANOVA, followed by post-hoc Tukey’s test (SPSS version 19), was used to compare effects of various chemicals on inhibition of growth or photosynthesis, among six species of algae.

Principal coordinates analysis (PCA) was used to visualize relationships among IC50s and EC50s of the six algal species exposed to the 5 zinc-containing compounds and distribution of expressed genes in *T*. *pseudonana* exposed to various concentrations for several durations. Responses gene markers in *T*. *pseudonana* were also evaluated by use of multivariate statistical analyses (PRIMER 6; Primer-E Ltd, Plymouth). All variables were normalized using Euclidean distances. Permutational multivariate analysis of variance (PERMANOVA) and analysis of similarity (ANOSIM) were conducted to infer if there were significant differences amongst the treatments of the three fixed factors (i.e., chemicals, exposure concentrations and time). Data were considered as statistically different when *p* < 0.05. Heatmaps of gene expression profiles were generated by Genesis software (Graz University of Technology, Austria).

### Data Availability

The datasets generated during and/or analysed during the current study are available from the corresponding author on reasonable request.

## Electronic supplementary material

Supplementary Information

## References

[1] Özgür Ü (2005). A comprehensive review of ZnO materials and devices. J. Appl. Phys..

[2] Serpone N, Dondi D, Albini A (2007). Inorganic and organic UV filters: their role and efficacy in sunscreens and suncare products. Inorg. Chim. Act..

[3] Wahab R (2011). Non-hydrolytic synthesis and photo-catalytic studies of ZnO nanoparticles. Chem. Eng. J..

[4] Wahab R (2013). Photocatalytic oxidation of acetaldehyde with ZnO-quantum dots. Chem. Eng. J..

[5] Wahab R (2015). Utilization of photocatalytic ZnO nanoparticles for deactivation of safranine dye and their applications for statistical analysis. Physica E.

[6] Dwivedi S (2014). Reactive oxygen species mediated bacterial biofilm inhibition via zinc oxide nanoparticles and their statistical determination. PLoS ONE.

[7] Hölken I (2016). Complex shaped ZnO nano- and microstructure based polymer composites: mechanically stable and environmentally friendly coatings for potential antifouling applications. Phys. Chem. Chem. Phys..

[8] Wahab R, Khan F, Mishra YK, Musarrat J, Al-Khedhairy AA (2016). Antibacterial studies and statistical design set data of quasi zinc oxide nanostructures. RSC Adv..

[9] Wahab R (2016). Self-styled ZnO nanostructures promotes the cancer cell damage and suppresses the epithelial phenotype of glioblastoma. Sci. Rep..

[10] Wahab R (2014). ZnO nanoparticles induced oxidative stress and apoptosis in HepG2 and MCF-7 cancer cells and their antibacterial activity. Colloids Surf. B Biointerfaces.

[11] Ahmad J, Wahab R, Siddiqui MA, Musarrat J, Al-Khedhairy AA (2015). Zinc oxide quantum dots: a potential candidate to detain liver cancer cells. Bioprocess Biosyst. Eng..

[12] Antoine TE (2012). Prophylactic, therapeutic and neutralizing effects of zinc oxide tetrapod structures against herpes simplex virus type-2 infection. Antiviral Res..

[13] Antoine TE (2016). Intravaginal zinc oxide tetrapod nanoparticles as novel immunoprotective agents against genital herpes. J. Immunol..

[14] Kathalewar M, Sabnis A, Waghoo G (2013). Effect of incorporation of surface treated zinc oxide on non-isocyanate polyurethane based nano-composite coatings. Prog. Org. Coat..

[15] Christian P, V der Kammer F, Baalousha M, Hofmann Th (2008). Nanoparticles: structure, properties, preparation and behaviour in environmental media. Ecotoxicol.

[16] Yin H, Casey PS, McCall MJ (2010). Surface modifications of ZnO nanoparticles and their cytotoxicity. J. Nanosci. Nanotechnol..

[17] Truong L, Saili KS, Miller JM, Hutchison JE, Tanguay RL (2012). Persistent adult zebrafish behavioral deficits results from acute embryonic exposure to gold nanoparticles. Comp. Biochem. Physiol. C.

[18] Suresh AK (2012). Cytotoxicity induced by engineered silver nanocrystallites is dependent on surface coatings and cell types. Langmuir.

[19] Grasset F (2003). Surface modification of zinc oxide nanoparticles by aminopropyltriethoxysilane. J. Alloy. Compd..

[20] Rohe B, Veeman WS, Tausch M (2006). Synthesis and photocatalytic activity of silane-coated and UV-modified nanoscale zinc oxide. Nanotechnol.

[21] Yin H, Tsuzuki T, Millington KR, Casey PS (2014). A comparative interlaboratory study on photocatalytic activity of commercial ZnO and CeO_2_ nanoparticles. J. Nanopart. Res..

[22] Osmond-McLeod M, Oytam Y, Osmond RIW, Sobhanmanesh F, McCall MJ (2014). Surface coatings protect against the *in vitro* toxicity of zinc oxide nanoparticles in human hepatic stellate cells. J. Nanomed. Nanotechnol..

[23] Wiench K (2009). Acute and chronic effects of nano- and non-nano-scale TiO_2_ and ZnO particles on mobility and reproduction of the freshwater invertebrate *Daphnia magna*. Chemosphere.

[24] Fröhlich E, Meindl C, Roblegg E, Griesbacher A, Pieber TR (2012). Cytotoxicity of nanoparticles is influenced by size, proliferation and embryonic origin of the cells used for testing. Nanotoxicology.

[25] Wu YL (2007). Surface modifications of ZnO quantum dots for bio-imaging. Nanotechnol.

[26] Bian SW, Mudunkotuwa IA, Rupasinghe T, Grassian VH (2011). Aggregation and dissolution of 4 nm ZnO nanoparticles in aqueous environments: influence of pH, ionic strength, size, and adsorption of humic acid. Langmuir.

[27] Garner KL, Keller AA (2014). Emerging patterns for engineered nanomaterials in the environment: a review of fate and toxicity studies. J. Nanopart. Res..

[28] Keller AA (2010). Stability and aggregation of metal oxide nanoparticles in natural aqueous matrices. Environ. Sci. Technol..

[29] Petosa AR, Jaisi DP, Quevedo IR, Elimelech M, Tufenkji N (2010). Aggregation and deposition of engineered nanomaterials in aquatic environments: role of physicochemical interactions. Environ. Sci. Technol..

[30] Degen A, Kosec M (2000). Effect of pH and impurities on the surface charge of zinc oxide in aqueous solution. J. Eur. Cera. Soc..

[31] Mallakpour S, Madani M (2012). Use of silane coupling agent for surface modification of zinc oxide as inorganic filler and preparation of poly(amide-imide)/zinc oxide nanocomposite containing phenylalanine moieties. Bull. Mater. Sci..

[32] Powell KJ (2013). Chemical speciation of environmentally significant metals with inorganic ligands. Part 5: the Zn^2+^  + OH^−^, Cl^−^, CO_3_^2−^, SO_4_^2−^, and PO_4_^3−^ systems (IUPAC Technical Report). Pure Appl. Chem..

[33] Kanel SR, Al-Abed SR (2011). Influence of pH on the transport of nanoscale zinc oxide in saturated porous media. J. Nanopart. Res..

[34] Gelabert A (2014). Uncoated and coated ZnO nanoparticle life cycle in synthetic seawater. Environ. Toxicol. Chem..

[35] Waalewijn-Kool PL, Ortiz MD, van Straalen NM (2013). Sorption, dissolution and pH determine the long-term equilibration and toxicity of coated and uncoated ZnO nanoparticles in soil. Environ. Pollut..

[36] Leung YH (2012). *Antibacterial activity of ZnO nanoparticles with a modified surface under ambient illumination*. Nanotechnol.

[37] Subbiahdoss G (2012). Magnetic targeting of surface-modified superparamagnetic iron oxide nanoparticles yields antibacterial efficacy against biofilms of gentamicin-resistant staphylococci. Acta. Biomaterialia..

[38] Peng X, Palma S, Fisher NS, Wong SS (2011). Effect of morphology of ZnO nanostructures on their toxicity to marine algae. Aquat. Toxicol..

[39] Lee DY, Fortin C, Campbell PGC (2005). Contrasting effects of chloride on the toxicity of silver to two green algae, *Pseudokirchneriella subcapitata* and *Chlamydomonas reinhardtii*. Aquat. Toxicol..

[40] Hiriart-Baer VP, Fortin C, Lee DY, Campbell PGC (2006). Toxicity of silver to two freshwater algae, *Chlamydomonas reinhardtii* and *Pseudokirchneriella subcapitata*, grown under continuous culture conditions: influence of thiosulphate. Aquat. Toxicol..

[41] Franklin NM, Stauber JL, Lim RP, Petocz P (2002). Toxicity of metal mixtures to a tropical freshwater alga (*Chlorella* sp.): the effect of interactions between copper, cadmium, and zinc on metal cell binding and uptake. Environ. Toxicol. Chem..

[42] Su G (2015). Comparison on the molecular response profiles between nano zinc oxide (ZnO) particles and free zinc ion using a genome-wide toxicogenomics approach. Environ. Sci. Pollut. Res. Int..

[43] Davis AK, Hildebrand M, Palenik B (2006). Gene expression induced by copper stress in the diatom *Thalassiosira pseudonana*. Eukaryotic. Cell..

[44] Shi X, Gao W, Chao SH, Zhang W, Meldrum DR (2013). Monitoring the single-cell stress response of the diatom *Thalassiosira pseudonana* by quantitative real-time reverse transcription-PCR. Appl. Environ. Microbiol..

[45] Wolfe-Simon F, Starovoytov V, Reinfelder JR, Schofield O, Falkowski G (2006). Localization and role of manganese superoxide dismutase in a marine diatom. Plant. Physiol..

[46] Shen C (2013). Relating cytotoxicity, Zinc ions and reactive oxygen in ZnO nanoparticles-exposed human immune cells. Toxicol. Sci..

[47] Bopp SK, Lettieri T (2007). Gene regulation in the marine diatom *Thalassiosira pseudonana* upon exposure to polycyclic aromatic hydrocarbons (PAHs). Gene.

[48] Carvalho RN (2011). Gene biomarkers in diatom *Thalassiosira pseudonana* exposed to polycyclic aromatic hydrocarbons from contaminated marine surface sediments. Aquat. Toxicol..

[49] Sirelkhatim A (2015). Review on zinc oxide nanoparticles: antibacterial activity and toxicity mechanism. Nano-Micro Lett..

[50] Díaz-Visurraga, J., Gutiérrez, C., von Plessing, C. & García, A. Metal nanostructures as antibacterial agents in *Science against microbial pathogens: communicating current research and technological advances* (ed. Méndez-Vilas, A.) 210-218 (Formatex, Badajoz, 2011).

[51] Raghupathi KR, Koodali RT, Manna AC (2011). Size-dependent bacterial growth inhibition and mechanism of antibacterial activity of zinc oxide nanoparticles. Langmuir.

[52] Aksmann A (2014). Time-dependent changes in antioxidative enzyme expression and photosynthetic activity of *Chlamydomonas reinhardtii* cells under acute exposure to cadmium and anthracene. Ecotoxicol. Environ. Safe..

[53] Stanier RY, Kunisawa R, Mandel M, Cohen-Bazire G (1971). Purification and properties of unicellular blue-green algae (Order Chroococcales). Bacteriol. Rev..

[54] Guillard, R. R. L. Culture of phytoplankton for feeding marine invertebrates. In Culture of Marine Invertebrate Animals (eds Smith, W. L., Chanley, M. H.) 29-60 (Plenum Press, 1975).

[55] OECD Guidelines for Testing of Chemicals – Freshwater Alga and Cyanobacteria, Growth Inhibition Test. Organisation for Economic Co-operation and Development (OECD): Paris, France (2011).

[56] Maxwell K, Johnson GN (2000). Chlorophyll fluorescence – a practical guide. J Exp Bot.

[57] Roháček K (2002). Chlorophyll fluorescence parameters: the definitions, photosynthetic meaning, and mutual relationships. Photosynthetica.

[58] Roháček K, Barták M (1999). Technique of the modulated chlorophyll fluorescence: basic concepts, useful parameters, and some applications. Photosynthetica.

[59] Armbrust EV (2004). The genome of the diatom *Thalassiosira pseudonana*: ecology, evolution and metabolism. Science.

[60] Yi AX, Leung PTY, Leung KMY (2014). Photosynthetic and molecular responses of the marine diatom *Thalassiosira pseudonana* to triphenyltin exposure. Aquat. Toxicol..

