# Supplementary material for: Physicochemical characteristics and toxicity of surface-modified zinc oxide nanoparticles to freshwater and marine microalgae
Source: Sci Rep. 2017 Nov 21;7:15909. doi: 10.1038/s41598-017-15988-0 (PMC5698420; doi:10.1038/s41598-017-15988-0)
Supplement: Supplementary file 1 — Supplementary Information [file 41598_2017_15988_MOESM1_ESM.pdf]

Supplementary Information

Physicochemical characteristics and toxicity of surface-modified zinc oxide nanoparticles to freshwater and marine microalgae

Mana M. N. Yung [a], Paul-Antoine Fougères [a,b], Yu Hang Leung [c], Fangzhou Liu [c], Aleksandra B. Djurišić [c], John P. Giesy [a,d,e] and Kenneth M. Y. Leung [a,f]*

[a] The Swire Institute of Marine Science and School of Biological Sciences, the University of Hong Kong, Pokfulam, Hong Kong, China
[b] Université de Bordeaux, Bordeaux, France
[c] Department of Physics, the University of Hong Kong, Pokfulam, Hong Kong, China
[d] Department of Veterinary Biomedical Sciences and Toxicology Centre, University of Saskatchewan, Saskatoon, SK, Canada
[e] State Key Laboratory of Pollution Control and Resource Reuse, School of the Environment, Nanjing University, Nanjing, China
[f] State Key Laboratory in Marine Pollution, City University of Hong Kong, Tat Chee Avenue, Kowloon, Hong Kong, China

*Corresponding author: Prof. Kenneth M. Y. Leung

Number of pages: 9

Zeta potentials

After stirring for 7 days, zeta potential of ZnO, ZnO-NPs, A-ZnO-NPs and D-ZnO-NPs suspensions in each of the 40 treatments (i.e., 2 culture media × 2 pH levels × 10 concentrations) was measured in triplicate (5 mL each) using a particle analyzer (Delsa Nano C, Beckman Coulter Inc., Germany).

The four test particles had mean zeta potentials within ± 30 mV in both BG-11 and f/2 algal culture media at pH 7 and 8 at all exposure concentrations (Fig. S1A-D). Therefore, the particles were unstable and they would aggregate readily in both culture media of pH 7 and 8 at the exposure concentrations. Such observations agreed with sizes of aggregations measured. Only exposure concentration had a significant effect on zeta potentials of test chemicals (Four-way ANOVA: $F_{5, 195} = 19.65$, $p < 0.001$), with zeta potentials between 30 and 100 mg/L more negative than those at 5 and 10 mg/L. However, the culture media, pH, size of the particles and the coating materials did not significantly affect zeta potentials of test particles.

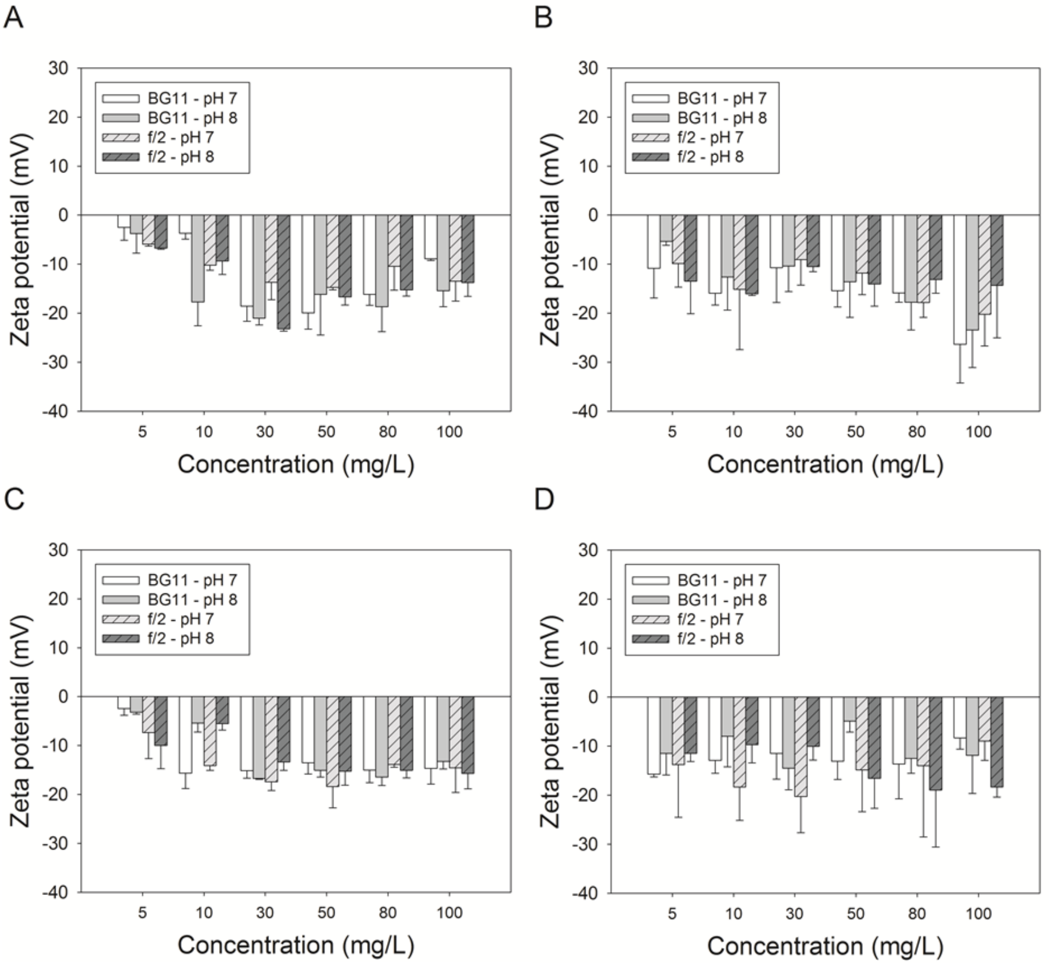

Figure S1. Mean zeta potential of: (A) ZnO; (B) ZnO-NPs; (C) A-ZnO-NPs and (D) D-ZnO-NPs in BG-11 and f/2 media at pH 7 and pH 8 after seven days of exposure at 25 °C (mean and 95% confidence interval, *n* = 3). Zeta potentials of particles at 0.1, 0.5, 1, and 3 mg/L were less than the limit of detection so such data are not shown.

Surface chemistries

Dry particles made of ZnO, ZnO-NPs, A-ZnO-NPs and D-ZnO-NPs were mixed with infrared grade KBr and pressed to form a pellet for Fourier transform infrared (FT-IR) spectroscopy analysis. FT-IR spectra of test chemicals were observed in the range of 400 to 4,000 cm^{-1} on Perkin Elmer (Spectrum Two, Waltham, MA, USA) FT-IR spectrometer.

An intense sharp peak at 400-600 cm^{-1} in the FT-IR spectra of ZnO, ZnO-NPs, A-ZnO-NPs and D-ZnO-NPs corresponded to Zn-O stretching vibration (Fig. S2A-D).

Absorption bands at 3,000-3,500 cm^{-1} and ~1,630 cm^{-1} were caused by the O-H stretching and bending of the absorbed water molecules from the environment, respectively[1]. A broader absorption peak at 3,000-3,500 cm^{-1} was observed in A-ZnO-NPs (Fig. S2C), this is merged with the N-H vibration. A shaper and intense peak at the same range was observed in D-ZnO-NPs (Fig. S2D), it merged with CH_2 and CH_3 vibrations. The weak peak at 2,900 cm^{-1} which was observed only in the two coated ZnO-NPs corresponded to CH_2 vibrations. The peaks at 800-1,100 cm^{-1} corresponded to Zn-O-Si stretching vibrations[2,3], indicating that the two coating materials were covalently bound to the surface of ZnO-NPs. The peak at 1,350-1,600 cm^{-1}, which corresponded to the stretching vibrations of the C-H, C-O and C=O, were found in FT-IR spectra of ZnO-NPs, A-ZnO-NPs and D-ZnO-NPs, rather than in that of ZnO. The C-H, C-O and C=O were corresponding carboxylic group which was possibly due to the presence of zinc acetate. Zinc acetate could be used as a raw material of ZnO-NPs synthesis[1].

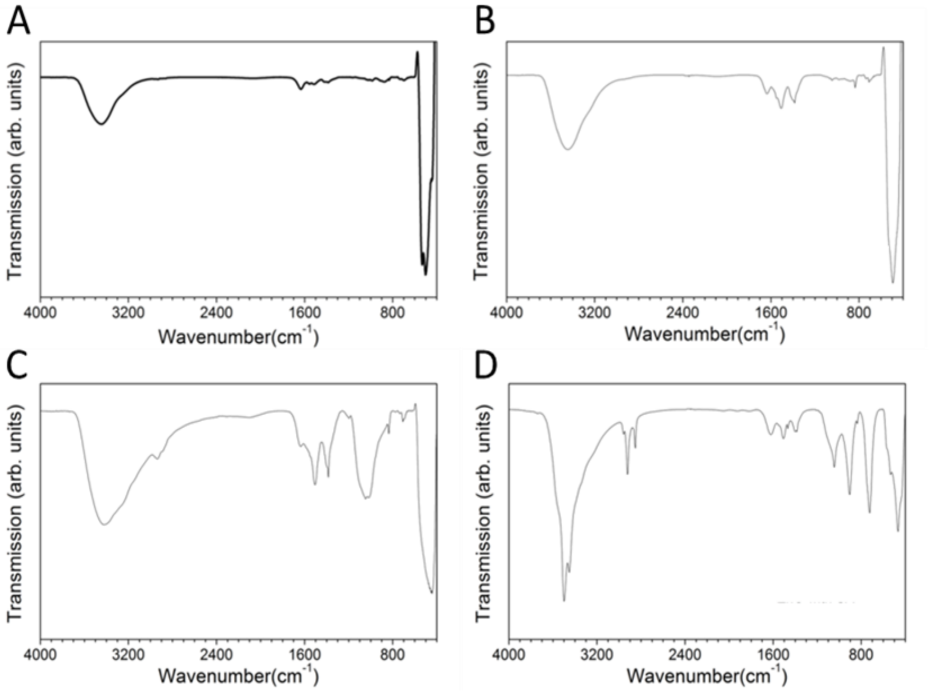

Figure S2. FTIR spectra of the dry powder of: (A) ZnO; (B) ZnO-NPs; (C) A-ZnO-NPs and (D) D-ZnO-NPs measured in the range of 400 to 4,000 cm^{-1}.

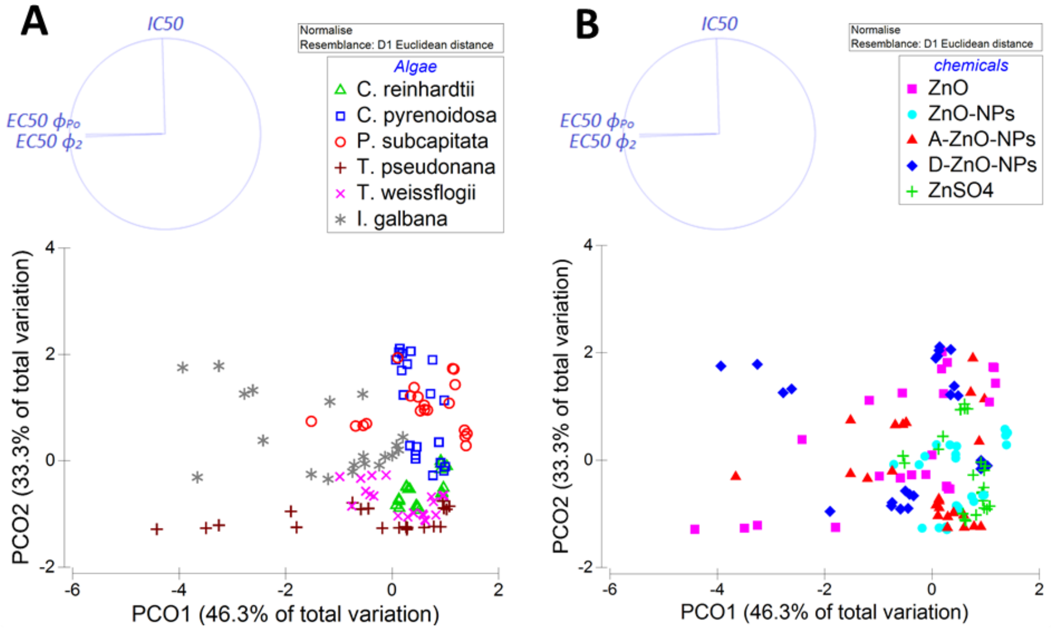

Figure S3. Principal component ordination of (A) the responses of the test microalgae and (B) the toxicity of the test chemicals based on normalized IC50 and EC50 values. Vectors showed the direction of the IC50 and EC50; the smaller the IC50 or EC50 values, the further down or right the dots located.

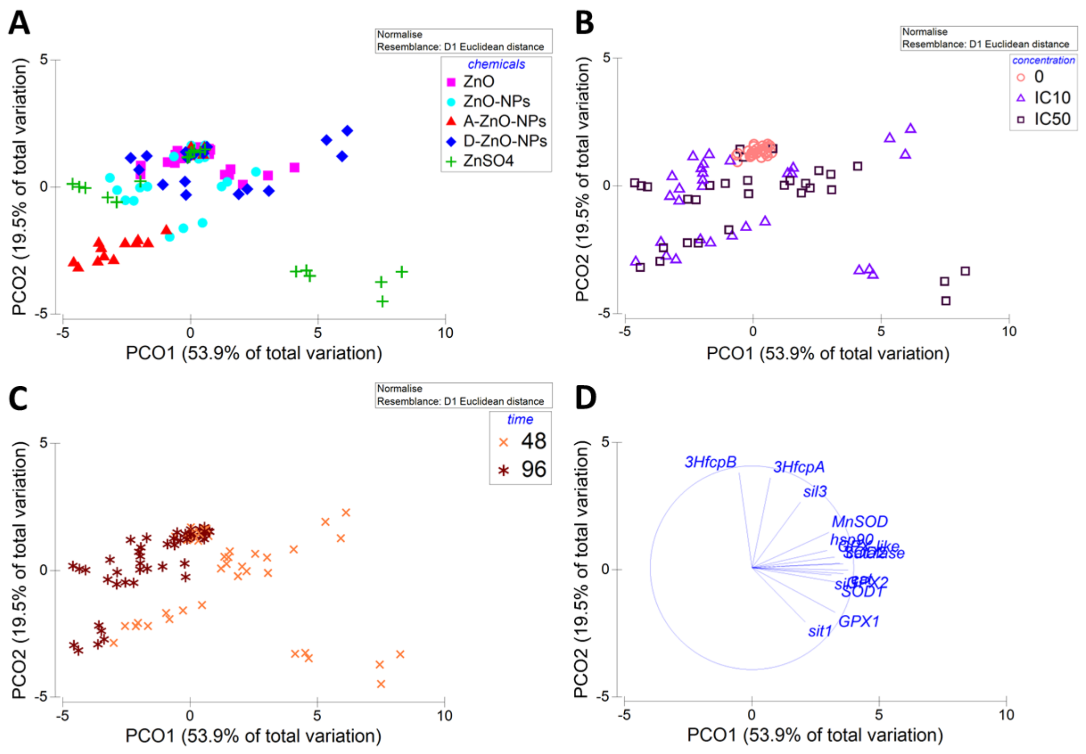

Figure S4. Principal component ordination plots of differentially expressed genes in the marine diatom *T. pseudonana* exposed to control and different (A) chemicals; (B) exposure concentrations; and at different (C) exposure time. (D) Vectors showed the direction of the expressed genes in *T. pseudonana*.

Table S1. Concentrations of ZnO, ZnO-NPs, A-ZnO-NPs, D-ZnO-NPs and $ZnSO_4$ to *Thalassiosira pseudonana* for gene expression study. Concentrations of dissolved zinc from the five zinc compounds were quantified by ICP-OES in f/2 medium at pH 8 (mean ± 95% confidence interval, *n* = 3).

	ZnO	ZnO-NPs	A-ZnO-NPs	D-ZnO-NPs	$ZnSO_4$
96-h IC10 (mg/L)	2.4	1.9	1.0	4.9	2.0
96-h IC50 (mg/L)	3.7	2.8	3.0	8.0	7.6
Measured concentrations of dissolved zinc in f/2 medium at pH 8					
96-h IC10 (mg/L of Zn)	0.09 ± 0.01	0.18 ± 0.02	0.08 ± 0.01	0.02 ± 0.01	0.35 ± 0.02
96-h IC50 (mg/L of Zn)	0.19 ± 0.01	0.21 ± 0.02	0.13 ± 0.01	0.08 ± 0.02	1.15 ± 0.02

Table S2. Primer sequences of the housekeeping gene and target genes.

Name of the gene	Abbrevia-tion	Primer sequences (5'→3')	References
Glyceraldehyde-3-phosphate dehydrogenase	*gapdh*	F: GGAGAAGGCCTCCATGCAT<br>R: TGGAGCCGAGATGACAACCT	4
Silaffin precursor 1	*sil1*	F: CCGTCACCCTCTCCTGAAAC<br>R: ATGGGAGCAGCGGTAATGG	4
Silaffin precursor 3	*sil3*	F: GGTGCAAAGAGTGCCAAGATG<br>R: GCTGCGTCCTCCGACTTTC	4
Silicon transporter 1	*sit1*	F: TTGCCGAGGATGCCTAAACTT<br>R: TGACGAGCTACTGCAGGTTCA	4
Fucoxanthin-chlorophyll a/c light harvesting protein	*3HfcpA*	F: CTCCCTCCAGGTTCCTGTTG<br>R: AGCGAGCTCAAGGAATCCAA	4
Fucoxanthin-chlorophyll a/c light harvesting protein	*3HfcpB*	F:AGTTCGATGAGGAGACCAAGCT<br>R: GGCACGTCCGTTGTTCAAC	4
Heat shock protein 90	*hsp90*	F: AGGCTCTTACGGCCGGGGCGGA<br>R: AAGACCCGCCAGCCTCGGAAGCC	5
Manganese superoxide dismutase	*MnSOD*	F: ATGAAAATCCATCATGATAAGCAT<br>R: TCCTCGCACGGGGACTCCTG	6
Superoxide dismutase	*SOD1*	F: AGTGAGCAAGTCGGCAGCGG<br>R: TAGCGTGCGGTGCGAGGTTG	5
Superoxide dismutase	*SOD2*	F: GCCACCACCATCGCTACACCC<br>R: GCCCGGCCTCCAAAGCATTCA	5
catalase	*cat*	F: GTTGATGATTCGGTTGGCTTGGC<br>R: AGTTGAGAGGTGCAAGACGGATG	7
catalase	*Catalase*	F: TGGGGGTGACTGCAGGGGCGA<br>R: AGCCGCCATCGATACCCCACCAGC	5

Table S2. (continued)

Name of the gene	Abbrevia-tion	Primer sequences (5’→3’)	References
Glutathione peroxidase	*GPX1*	F: CAAAGGCGACGTGCTATGCGTC<br>R: GGCTCCTGAGCTCCAAACTGATT	7
Glutathione peroxidase	*GPX2*	F: TTCGGAGCAACTTTCCAGAGGTG<br>R: TGCCCATAACTCTTGACAGCCCT	7
Glutathione peroxidase	*GPX-like*	F: AAACTCCACCGCAAATACAAATCC<br>R: CACGTAATCCTAGGAGGACCG	7

References

1. Song, H., Yang, H. & Ma, X. A comparative study of porous ZnO nanostructures synthesized from different zinc salts as gas sensor materials. *J. Alloy. Compd.* **578**, 272-278 (2013).
2. Kathalewar, M., Sabnis, A. & Waghoo, G. Effect of incorporation of surface treated zinc oxide on non-isocyanate polyurethane based nano-composite coatings. *Prog. Org. Coat.* **76**, 1215-1229 (2013).
3. Tang, E. & Dong, S. Preparation of styrene polymer/ZnO nanocomposite latex via miniemulsion polymerization and its antibacterial property. *Colloid. Polym. Sci.* **287**, 1025-1032 (2009).
4. Bopp, S. K. & Lettieri, T. Gene regulation in the marine diatom *Thalassiosira pseudonana* upon exposure to polycyclic aromatic hydrocarbons (PAHs). *Gene* **396**, 293-302 (2007).
5. Shi, X., Gao, W., Chao, S. H., Zhang, W. & Meldrum, D.R. Monitoring the single-cell stress response of the diatom *Thalassiosira pseudonana* by quantitative real-time reverse transcription-PCR. *Appl. Environ. Microbiol.***79**, 1850-1858 (2013).
6. Wolfe-Simon, F., Starovoytov, V., Reinfelder, J. R., Schofield, O. & Falkowski, G. Localization and role of manganese superoxide dismutase in a marine diatom. *Plant Physiol.* **142**, 1701-1709 (2006).
7. Davis, A. K., Hildebrand, M. & Palenik, B. Gene expression induced by copper stress in the diatom *Thalassiosira pseudonana*. *Eukaryotic Cell* **5**, 1157-1168 (2006).
